# Insight of Genus *Corynebacterium*: Ascertaining the Role of Pathogenic and Non-pathogenic Species

**DOI:** 10.3389/fmicb.2017.01937

**Published:** 2017-10-12

**Authors:** Alberto Oliveira, Leticia C. Oliveira, Flavia Aburjaile, Leandro Benevides, Sandeep Tiwari, Syed B. Jamal, Arthur Silva, Henrique C. P. Figueiredo, Preetam Ghosh, Ricardo W. Portela, Vasco A. De Carvalho Azevedo, Alice R. Wattam

**Affiliations:** ^1^Molecular and Cellular Laboratory, General Biology Department, Federal University of Minas Gerais, Belo Horizonte, Brazil; ^2^Center of Genomics and System Biology, Federal University of Pará, Belém, Brazil; ^3^Aquacen, National Reference Laboratory for Aquatic Animal Diseases, Federal University of Minas Gerais, Belo Horizonte, Brazil; ^4^Department of Computational Science, Virginia Commonwealth University, Richmond, VA, United States; ^5^Laboratory of Immunology and Molecular Bióloga, Health Sciences Institute, Federal University of Bahiaa, Salvador, Brazil; ^6^Biocomplexity Institute of Virginia Tech, Virginia Tech, Blacksburg, VA, United States

**Keywords:** *Corynebacterium*, bacterial genomics, bacterial biochemistry, pathogenesis related genes, biotechnology of microorganisms

## Abstract

This review gathers recent information about genomic and transcriptomic studies in the *Corynebacterium* genus, exploring, for example, prediction of pathogenicity islands and stress response in different pathogenic and non-pathogenic species. In addition, is described several phylogeny studies to *Corynebacterium*, exploring since the identification of species until biological speciation in one species belonging to the genus *Corynebacterium*. Important concepts associated with virulence highlighting the role of Pld protein and Tox gene. The adhesion, characteristic of virulence factor, was described using the sortase mechanism that is associated to anchorage to the cell wall. In addition, survival inside the host cell and some diseases, were too addressed for pathogenic corynebacteria, while important biochemical pathways and biotechnological applications retain the focus of this review for non-pathogenic corynebacteria. Concluding, this review broadly explores characteristics in genus *Corynebacterium* showing to have strong relevance inside the medical, veterinary, and biotechnology field.

## Introduction

This review is limited to a general discussion of the genus *Corynebacterium* and its species. It includes specific information on the genes that are found in pathogenic strains compared to those that are non-pathogenicity. Various studies describe these bacteria as gram-positive (Hard, 1975; Dorella et al., 2006) but with shape, oxygen requirement, and environment of preference divergent dependent on specie, that constitute a very heterogeneous group. The genus *Corynebacterium*, which currently has more than 110 validated species, is highly diversified. It includes species that are of medical, veterinary, or biotechnological relevance (Bernard, 2012; Soares et al., 2013; Eikmanns and Blombach, 2014). In an examination of the diverse group to which these bacteria belong, this study describes the structural organization, function, and dynamism of genome. This review It also explores some important process, such as cell division, in order to improve the understanding of this genus, the pathogenic processes that is directly associated with several diseases and symptoms and some biochemical pathways associated with biotechnology industry explored by the non-pathogenic species. In addition, this work explores genes and pathways that may play a role in pathogenesis, and compare them to those found in non-pathogenic strains. Table 1 provides a list of the main species and important information about them, showing some species studied currently.

**Table 1:** A list of well-known *Corynebacterium* species and some of the significant features that define them.

**Taxonomy**	**Pathogenisis**	**Number of genomes**	**Oxygen requirement**
*C. accolens* (Ang and Brown, 2007; Wong et al., 2010)	Pelvic Osteomyelitis; Granulomatous Mastitis	2	Facultative
*C. ammoniagenes*		3	Anaerobic
*C. amycolatum*	Endocarditis; Sepsis	3	Aerobic
*C. argentoratense* (Riegel et al., 1995)	Tonsillitis	3	Unknown
*C. atypicum*		1	Facultative
*C. aurimucosum* (Lo et al., 2015)	Urinary tract infection	15	Facultative
*C. auriscanis* (Collins et al., 1999)		1	Aerobic
*C. bovis* (Burr et al., 2012)	Hyperkeratosis; Mastitis	1	Facultative
*C. callunae* (Persicke et al., 2015)	Non-pathogenic|Amino acid production	2	Aerobic
*C. camporealensis*	Mastitis	2	Facultative
*C. capitovis* (Collins et al., 2001)		1	Anaerobic
*C. casei*	Non-pathogenic|Food production	2	Facultative
*C. caspium*		1	Facultative
*C. ciconiae*		1	Anaerobic
*C. crenatum* (Xu et al., 2012; Su et al., 2015)	Non-pathogenic|Amino acid production	2	Aerobic
*C. deserti*		1	Aerobic
*C. diphtheriae*	Diphtheria	86	Aerobic
*C. doosanense*		2	Facultative
*C. durum* (Riegel et al., 1997; James et al., 2011)		2	Facultative
*C. efficiens* (Odeniyi et al., 2017)	Non-pathogenic|Laccases and Amino acid production	2	Facultative
*C. epidermidicanis* (Frischmann et al., 2012)		1	Facultative
*C. falsenii*		2	Facultative
*C. freiburgense*		1	Facultative
*C. freneyi* (Auzias et al., 2003; Funke and Frodl, 2008)	Bacteremia	1	Anaerobic
*C. genitalium*		1	Facultative
*C. glucuronolyticum*		2	Facultative
*C. glutamicum*	Non-pathogenic|Food and Amino acid production	28	Facultative
*C. glycinophilum*	Non-pathogenic|Amino acid production	1	Aerobic
*C. halotolerans* (Rückert et al., 2012)	Non-pathogenic	3	Aerobic
*C. humireducens*		2	Facultative
*C. imitans*		1	Unknown
*C. jeikeium*	Nosocomial infections	24	Facultative
*C. kroppenstedtii*		4	Facultative
*C. kutscheri*	Lung abscess	1	
*C. lactis* (Antunes et al., 2015)		1	Facultative
*C. lipophiloflavum*		1	
*C. lubricantis*		1	
*C. marinum*		1	Facultative
*C. maris*		1	Aerobic
*C. massiliense*		1	
*C. mastitidis*	Subclinical mastitis	1	
*C. matruchotii*	Oral infections	2	Aerobic
*C. minutissimum* (Granok et al., 2002)	Erythrasma	3	Facultative
*C. mustelae* (Funke et al., 2010)		1	Aerobic
*C. nuruki* (Shin et al., 2011)	Non-pathogenic|Alcohol fermentation	1	Aerobic
*C. pilosum*	Cystitis	2	
*C. propinquum* (Abdolrasouli and Roushan, 2013)	Urethritis	1	Facultative
*C. pseudodiphtheriticum* (Manzella et al., 1995; Martaresche et al., 1999)	Nosocomial Pneumonia; Bronchitis	2	
*C. pseudotuberculosis*	Caseous Lymphadenitis	71	Facultative
*C. pyruviciproducens* (Tong et al., 2010, 2012)	Non-pathogenic|Pyruvic acid and ovalbumin production	1	Facultative
*C. renale* (Honda and Yanagawa, 1973)	Cystitis; Pyelonephritis	1	Facultative
*C. resistens* (Schröder et al., 2012)	Bacteremia	1	
*C. riegelii* (Funke et al., 1998)		1	Facultative
*C. simulans* (Wattiau et al., 2000)		3	Facultative Anaerobic
*C. singulare*		1	Facultative
*C. sp. ATCC*		124	
*C. sputi*		1	Anaerobic
*C. stationis*		4	
*C. striatum* (Sheldon and Coudron, 1990)	Endocarditis	3	
*C. terpenotabidum* (Rückert et al., 2013)	Non-pathogenic|Squalene production	1	Aerobic
*C. testudinoris*		1	
*C. timonense* (Henrique et al., 2011; Merhej et al., 2016)		2	Facultative
*C. tuberculostearicum*		1	Anaerobic
*C. tuscaniense*		1	
*C. ulcerans* (De Zoysa et al., 2005b)	Pharyngeal Diphtheria	17	
*C. ulceribovis*	Ulceration	1	Anaerobic
*C. urealyticum*	Urinary tract infection	3	Aerobic
*C. ureicelerivorans*	Septicemia	1	Facultative
*C. uterequi*		1	Facultative
*C. variabile* (Schröder et al., 2011)	Non-pathogenic|Food production	3	Aerobic
*C. vitaeruminis* (Al-Dilaimi et al., 2014)		3	Facultative
*C. xerosis*		2	

*The source of information were obtained by PATRIC Database (Wattam et al., 2014) and updated with new bibliographical sources*.

## Genomics and transcriptomics

Next-generation high-throughput DNA sequencing techniques (Ansorge, 2009) combined with new computational advances in assembly, annotation, and comparative analysis have resulted in an explosion of data available for research biologists. This wealth of information has pushed the field of genomics beyond the examination of sequences from single isolates to the ability to look across hundreds of genomes simultaneously. This new comparative ability adds significant advantages, not only for looking at differences that may exist within specific isolates, but can also shed light on the evolutionary process at broad taxonomic levels (Alfo and Lindblad-toh, 2013). This increases our understanding of chromosomal structure and the role played by horizontal transfer (Ochman et al., 2000). Broad comparisons between bacteria that cause disease with their closest relatives can highlight the genes and sequences that play a role in pathogenicity (Cohen et al., 2015; Papaventsis et al., 2017).

The genus *Corynebacterium* includes a diverse number of species and strains. As of April 2017, the PATRIC database (Wattam et al., 2017) had 466 genomes from 83 separate species (Supplementary Table 1). Some characteristics that unite these different species include having a single, circular chromosome, originally projected to range from 2.5 to 3.1 Mb in size (Dorella et al., 2006), and a high GC content (Pinto et al., 2014). A close examination of the current data shows that the size of the genomes within *Corynebacterium* range from 1.84 (*C. caspium* DSM 44850, ARBM00000000.1) to 15.8 Mb (*C. striatum* strain BM4687, CTEG00000000.1). There is also a striking range in the GC content, from a low of 10.7 (*C. striatum* strain BM4687, CTEG00000000.1) to 74.7% (*C. sphenisci* DSM 44792, CP009248.1). Generally, *Corynebacterium* lack plasmids, although there are several strains that have 1 or 2 plasmids as part of their genome (Supplementary Table 1). They also vary in the number of coding sequences (CDSs) that they have. An examination of all available genomes (Wattam et al., 2017) shows an average of 2480.6 CDSs, with *C. caspium* DSM 44850 as the strain with the fewest called genes (1647) and *C. aurimucosum* strain 118_CAUR have the most (9489). These various genomes have been isolated both from environmental sources as well as from individual hosts, and has several important pathogenic members that include *C. diptheriae, C. pseudotuberculosis*, and *C. ulcerans*.

Several studies have looked at the phylogeny of Corynebacterium, but only across a limited number of strains. A study (Pascual et al., 1995) did the first examination using 16S rRNA sequences and gave a direct comparison of Corynebacterium to its closest relatives, that included *Mycobacterium, Gordona, Rhodococcus*, and *Norcardi*. This was followed by a study of 56 species (Khamis et al., 2004) that looked at examined phylogeny two separate genes (16S and *rpoS*) to try to identify the best method to define *Corynebacterium* species. A later study by the same authors showed that a partial rpoB sequence provided the best method for identification of corynebacteria (Khamis et al., 2005). Gao and Gupta expanded the number of sequences used to build a tree when they created looked at patterns across the phylum Actinobacteria, including 14 *Corynebacterium* isolates, using the RpoB, RpoC, and gyrase B proteins joined into a single consensus sequence (Gao and Gupta, 2012). None of the studies showed the same branching patterns, but there were similarities across each study, with the pathogenic species *C. diptheriae, C. pseudotuberculosis*, and *C. ulcerans* all grouped together into a “pathogenic clade”. Including more genes in a similarity matrix also grouped these three species together (Soares et al., 2013). A strong phylogenetic analysis across this genus using a larger analysis including many more genes would resolve some of the differences seen between the earlier studies, and provide more insight into the evolution of the genus. Particularly, Phylogenetic approaches can be applied as methods for the identification of new species formation. It was a goal of a recent work showing events of biological speciation (Oliveira et al., 2016), among strains of the *C. pseudotuberculosis* species, in which it was proposed these strains could be under anagenesis process checking information such as: presence of biovar equi or ovis, evolutionary divergences, number of transition, and transversion substitutions and protein structure analysis.

Phylogenetic studies typically examined genes that are shared across all member, but several papers have described horizontal gene transfer as playing an important role in the evolution of *Corynebacterium*, particularly in terms of pathogenicity and virulence. A study of *C. pseudotuberculosis* identified seven islands that appeared to be linked to the pathogenicity of this species (Rocha et al., 2011). These islands included genes that have traditionally been associated with virulence, and these included iron uptake, secreted toxins, fimbrial subunits, and adhesion factors. This study was expanded, and six more islands were detected (Soares et al., 2013). Examination of *C. diptheriae* also identified a number of pathogenicity islands (Trost et al., 2012), and these islands, which include the *tox* gene (Efstratiou et al., 2003), CRISPR (Clustered Regularly Interspaced Short Palindromic Repeats) regions (Vívian et al., 2012), and pilus gene clusters (Trost et al., 2012), are suggested to be a driving force in evolution and pathogenesis of this species (Sangal and Hoskisson, 2016). While most of the studies involving horizontal transfer have examined pathogenic strains, it has also been identified in non-pathogenic strains (Kalinowski et al., 2003). Surprisingly, an incidence of horizontal transfer of a region containing a prophage was found in *C. glutamicum* ATCC 13032, a non-pathogenic strain of industrial importance, whose source seemed to be *C. diptheriae* (Kalinowski et al., 2003). This begs the question of whether the island is ancestral and lost in some members of *Corynebacterium*, or if it is a true transfer. A detailed genomic analysis across the genome could shed light on these types of incidents.

Studies that examine transcriptome in this genus are somewhat sparse, the majority of them examining the gene expression in *C. glutamicum*, which is not surprising considering the role it plays in the industrial production of amino acids (Lee et al., 2016). There are a number of microarray studies, a few that use RNA-Seq to examine transcription (Jiyoon et al., 2013; Mentz et al., 2013; Neshat et al., 2014; Sun et al., 2016; Albersmeier et al., 2017) and a single experiment that uses CHiP-seq (Jungwirth et al., 2013). There is a single study that looks beyond *C. glutamicum*. In order to analyze strains from *C. glutamicum* by RNA-seq in different degree of dissolved oxygen (DO) conditions in bioreactors, a recent work (Sun et al., 2016) showed how the DO effect could influence in the energy metabolism in this species. At a low DO rate, the glycolysis pathway was up-regulated effecting the over-production of amino acids, such as cysteine, arginine, and valine. This work has provided good ways to improve the oxygen acceptance by *C. glutamicum* in order to enhance the heterologous protein expression and amino acid production. In addition, (Pinto et al., 2014) a work examined gene expression patterns that were altered by three different conditions such as acid, osmotic, and thermal shock stress of abiotic stress in *C. pseudotuberculosis*. Several genes were observed to be at the minimum a 2-fold change in expression levels, which could describe important genes associated with infectious function associated in virulence, defense against adhesion, regulation, and oxidative stress.

## Cell division

**Figure 1:**
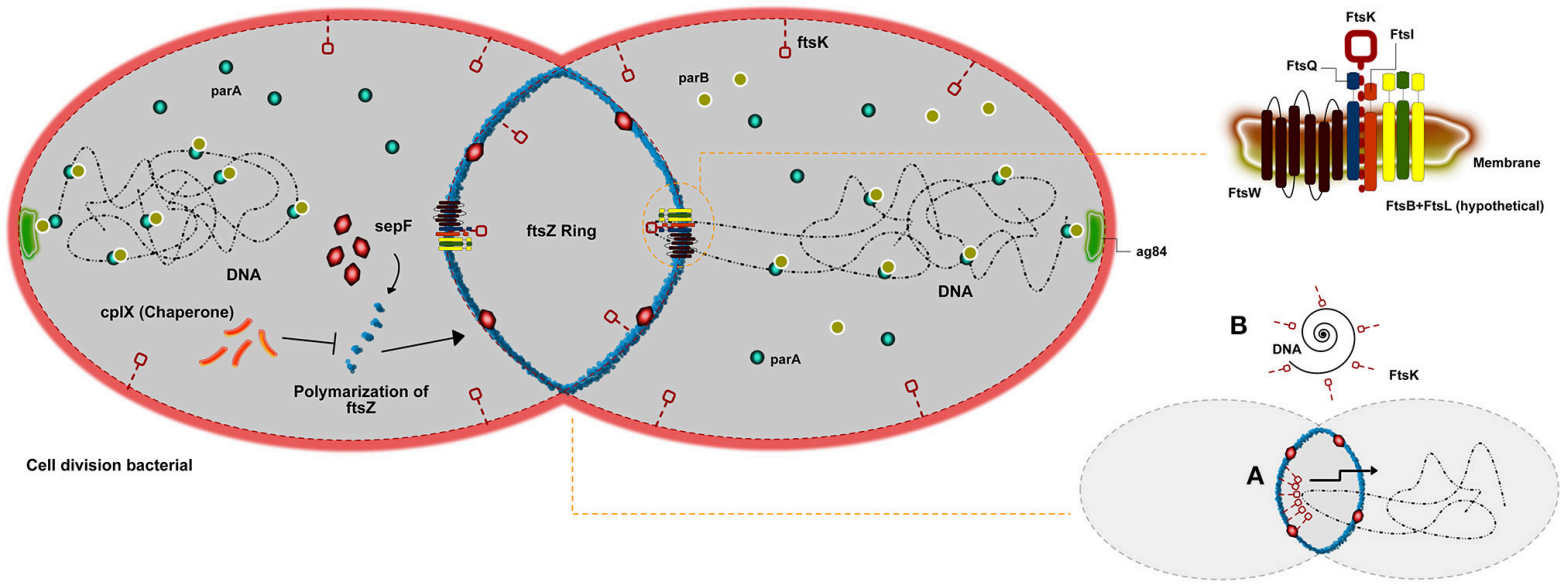
Scheme of cell division in *Corynebacterium* genus. An overview of cell division in *Corynebacterium* describing the main proteins involved. The FtsZ ring formation being regulated by clpX or shaped by sepF. The set of fts proteins associated with FtsZ (FtsQ, FtsK, FtsW, and FtsI) able to organize cell division, and finally the Par system that is described as important for chromosome segregation. In addition, a short scheme illustrating how the FtsW can help on the chromosome segregation.

Bacterial reproduction can be understood by ordered process in which cells increase in volume, duplicate genetic material, and isolate the newly formed DNA molecules (chromosomes) before detach the DNA to two daughter cells. Divisome is a known bacterial event resulting from the macromolecular machinery promoted by the order of DNA replication, chromosome segregation, and cytokinesis (Wu and Errington, 2011). A principal player involved in bacterial cell division in all bacteria is FtsZ. This protein initiates the polymerization of tubulin into a ring-like structure to start the divisome (Goehring and Beckwith, 2005). Some studies have demonstrated that FtsZ works in a cooperative assembly, creating in a polymeric form, such as rings, bundles, tubules, and sheets that were observed depending on the experimental conditions used (Romberg et al., 2001; Caplan and Erickson, 2003; Chen et al., 2005). A pivotal factor of the Z ring cluster is the interaction between FtsZ and the cell membrane. However, FtsZ is a protein unable to interacting with the membrane alone, until more recent results. Because of this, all proposed models for Z ring assembly depend upon a setup of other molecules in order to attach it to the membrane (Erickson, 1997, 2001; Goehring and Beckwith, 2005; Dajkovic et al., 2008) (Figure 1). A study (Pichoff and Lutkenhaus, 2002) in *Escherichia coli* proposed that two proteins, FtsA and ZipA, work together in order to anchor FtsZ to the membrane. This evidence FtsZ-ZapA-ZipA could be observed in other bacterial types, such as *Bacillus subtilis* (Gamba et al., 2009). However, species from the genus *Corynebacterium* do not present sequences of either FtsA or ZipA in their genomes nor do they present homologs to them (Letek et al., 2007; Fiuza et al., 2008), which suggests that FtsZ assembly is accomplished by another protein or set of proteins. A study, using fluorescence and differential interference contrast micrographs, describes how FtsZ is able to interact with a setup of proteins in *Corynebacterium*, such as FtsQ, FtsW, FtsK, and FtsI. These proteins form a cluster of molecules on the membrane that recruit FtsZ in order to promote cytokinesis (Hale and De Boer, 2002). Although FtsA is essential for viability in *E. coli*, it is absent in *Corynebacterium* species. Another FtsZ-interacting protein, SepF (also named YlmF), is able to supersede FtsA in genus *Corynebacterium*, suggesting that FtsA and SepF have overlapping functions (Ishikawa et al., 2006). In *B. subtilis*, is mentioned that SepF protein is not pivotal for cell division, in which its overproduction could balance for the lack of FtsA (Ishikawa et al., 2006). However, another work exploring the role of SpeF in *Mycobacterium*—A homologous genus to *Corynebacterium*—showed it is indeed essential for division (Gola et al., 2015). Nevertheless, the complete absence of SepF can harm the normal morphology of cells. The absence of both proteins is described as lethal to the cell (Ishikawa et al., 2006). The setup of proteins FtsQ-FtsW-FtsK-FtsL described, are membrane proteins with transmembrane domains. The function of some of these molecules on septum-formation is still not completely clear, although it has been proposed to be associated with shape formation, elongation, division, and sporulation (Alvarez et al., 1997; Mohammadi et al., 2011). Particularly, FtsK shows to act at the cell division septum in order to translocate DNA into the correct cellular compartment. Differently, associated regulation proteins cell division under stress conditions play a role on inhibits Z-ring formation are mentioned as highly conserved in a high amount of bacteria. In *Corynebacterium* the protein Clpx, that is a ATP-dependent protease, has been proposed inhibiting FtsZ polymer dynamics, either through degradation of the FtsZ monomers and polymers (Camberg et al., 2009) or by disassembling FtsZ polymers (Sugimoto et al., 2010). Chromosome segregation can also be explained by the genes parA and parB, which were previously described as important in cell control (Ginda et al., 2013). The same study showed that parA is able to interact with the polar growth determinant DivIVA, required for polar localization of the chromosome during sporulation (Sieger and Bramkamp, 2015), which in *Corynebacterium* is ag84. It is suggested that the Par system present an additional function in the replication procedure of DNA and too in the precision of the divisions sites, regarding the ParA-ParB Interactions already described during plasmid partitioning (Volante and Alonso, 2015). In addition, DNA damage can promote the inhibition of Z-ring formation, preventing the production of offspring with damaged genomes. Two proteins are responsible to control a response induced under such conditions. When activated, the RecA protein is able to stop the DNA-binding capacity in connecting with LexA, causing its activation and leading to DNA autocatalytic cleavage.

## Main features of pathogenic species

### Associate diseases and epidemiology

Among all *Corynebacterium* species isolated from humans, a group of potentially toxigenic microorganisms associated to different infectious processes stands out: *Corynebacterium diphtheriae, Corynebacterium ulcerans*, and *Corynebacterium pseudotuberculosis*. These species can produce potent exotoxins, i.e., diphtheria toxin (DT) and/or phospholipase D. These exotoxins can be associated with a large number of diseases, for example diphtheria and caseous lymphadenitis (Tauch and Sandbote, 2014). The species *C. diphtheriae* is the most significant pathogen of this group, in which it was the primary cause of diphtheria, a disease that is almost eradicated from developed countries due to a universal vaccine that targets the diphtheria toxin (Galazka and Dittmann, 2000; Wagner et al., 2012). This toxin can also be produced by *C. ulcerans* and *C*. *pseudotuberculosis*. These species are zoonotic agents, historically thought to induce diseases in humans after contact with contaminated animals (Wagner et al., 2010; Dias et al., 2011; Bernard, 2012). Other *Corynebacterium* species associated with animal diseases include: *Corynebacterium amycolatum, Corynebacterium aquilae, Corynebacterium auriscanis, Corynebacterium bovis, Corynebacterium camporealensis, Corynebacterium canis*, and others (Tauch and Sandbote, 2014).

### Diphtheria

Diphtheria is a notifiable disease and needs public health response to limit spread among patients (Bernard, 2005). This is a toxin-mediated disease which affects the region of the respiratory tract, generally characterized by the presence of an inflammatory pseudomembrane on the tonsils, oropharynx, and pharynx manifesting sore throat, fever, and some cases able to achieve death (Hadfield et al., 2000). The classic symptoms associated with the disease are lymphadenitis, dysphagia, headaches, sore throat, low-grade fever, and malaise (Bernard, 2005). This disease can cause kidney disease, neuritis, hearing loss, and myocarditis (Funke et al., 1997; Roux et al., 2004). The most common type of diphtheria can be caused by toxigenic strains of *C. diphtheriae, C. ulcerans*, and *C. pseudotuberculosis*. Zoonotic infection in humans is generally caused by *C. ulcerans* and *C. pseudotuberculosis*, whereas *C. diphtheriae* appears to be generally human specific (Wagner et al., 2010; Sangal et al., 2014). These are the only species in the genus capable of harboring a bacteriophage that carries DT-specific *tox* gene and potentially express DT (Holmes, 2000). Tox gene sequence is described highly conserved which confers the quality of toxoid vaccine response. The regulation of tox gene transcription is made by the DtxR regulon, which controls the balance of iron homeostasis (De Zoysa et al., 2005a; Trost et al., 2012). Some strains of *C. diphtheria, C. ulcerans*, or *C. pseudotuberculosis* lack *tox* gene, or have *tox* gene but no DT detectable, are considered to be non-toxigenic (Efstratiou and George, 1999; Sangal and Hoskisson, 2016). Even considered as non-toxigenic, these strains, especially *C. diphtheriae*, can cause diseases, like pharyngitis and tonsillitis, endocarditis, septic arthritis, and osteomyelitis, in certain vulnerable populations (Funke and Bernard, 2011; Sangal and Hoskisson, 2016).

### Caseous lymphadenitis

**Figure 2:**
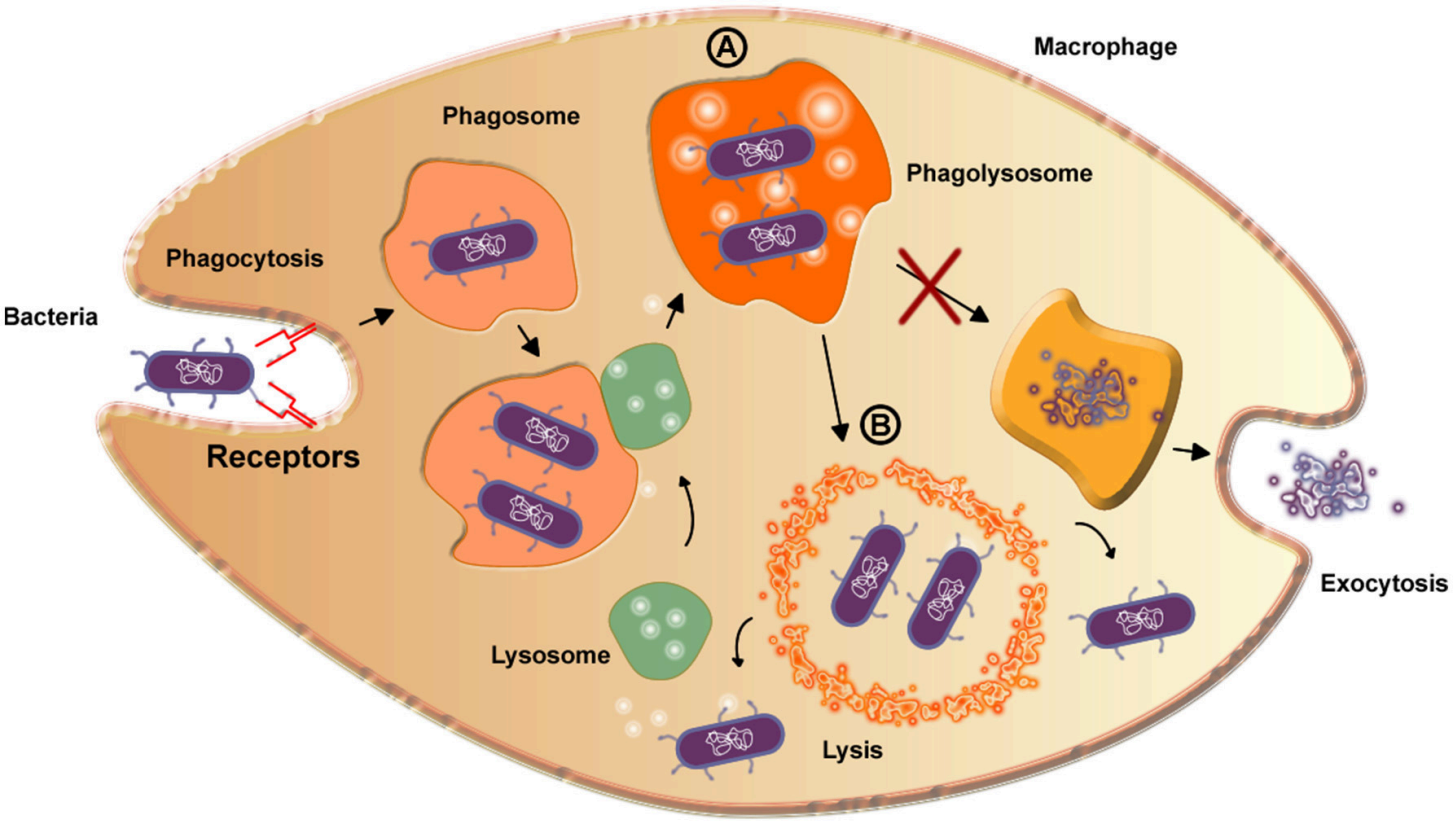
Scheme detailing the bacterial reproduction process in macrophages. A—The formed phagolysosome increases pH value and thereby increases PLD expression. Curiously, the phagolysosome is not able to digest the bacteria due to the presence of mycolic acid on the surface of the bacteria, inhibiting exocytosis. B—The high amount of PLD is enough to destroy the phagolysosome membrane by lysis, exposing the bacteria to the cytoplasm, in which they are able to survive and reproduce.

The etiologic agent of caseous lymphadenitis is *C. pseudotuberculosis*. This disease is common in small ruminant populations throughout the world and can cause a significant economic impact for producers. *C. pseudotuberculosis* infection in humans is not common, but has been reported on several occasions, making the disease a potential zoonosis (Peel et al., 1997; Dorella et al., 2006; Fontaine and Baird, 2008; Mills et al., 2009). The infectious process occurs mainly in animals, with clinical symptoms or asymptomatic, and the bacterial transmission that promotes the disease is usually spread by superficial wounds (Windsor and Bush, 2016). The primary virulence factor of *C. pseudotuberculosis* is Phospholipase D (Dorella et al., 2006), an exotoxin that promotes the hydrolysis and degradation of sphingomyelin in endothelial cell membranes (Figure 2), increasing the vascular permeability, which then facilitates to the spread and persistence of the bacterium in the host (Williamson, 2001).

There are two biovars of *C. pseudotuberculosis*: biovar ovis and biovar equi. The biovar ovis strains are responsible for causing infection mainly in sheep and goats. The principal characteristics of caseous lymphadenitis in these animals are the formation of caseous necrosis on the lymphatic glands or abscess formation in superficial lymph nodes and subcutaneous tissues (Baird and Fontaine, 2007). *C. pseudotuberculosis* biovar equi also can infect horses manifesting different disease patterns like external abscesses, ulcerative lymphangitis of the limbs, and some internal organs can be affects as called the visceral form Pratt et al. (2005).

## Virulence factors

### Adhesion mechanisms

**Figure 3:**
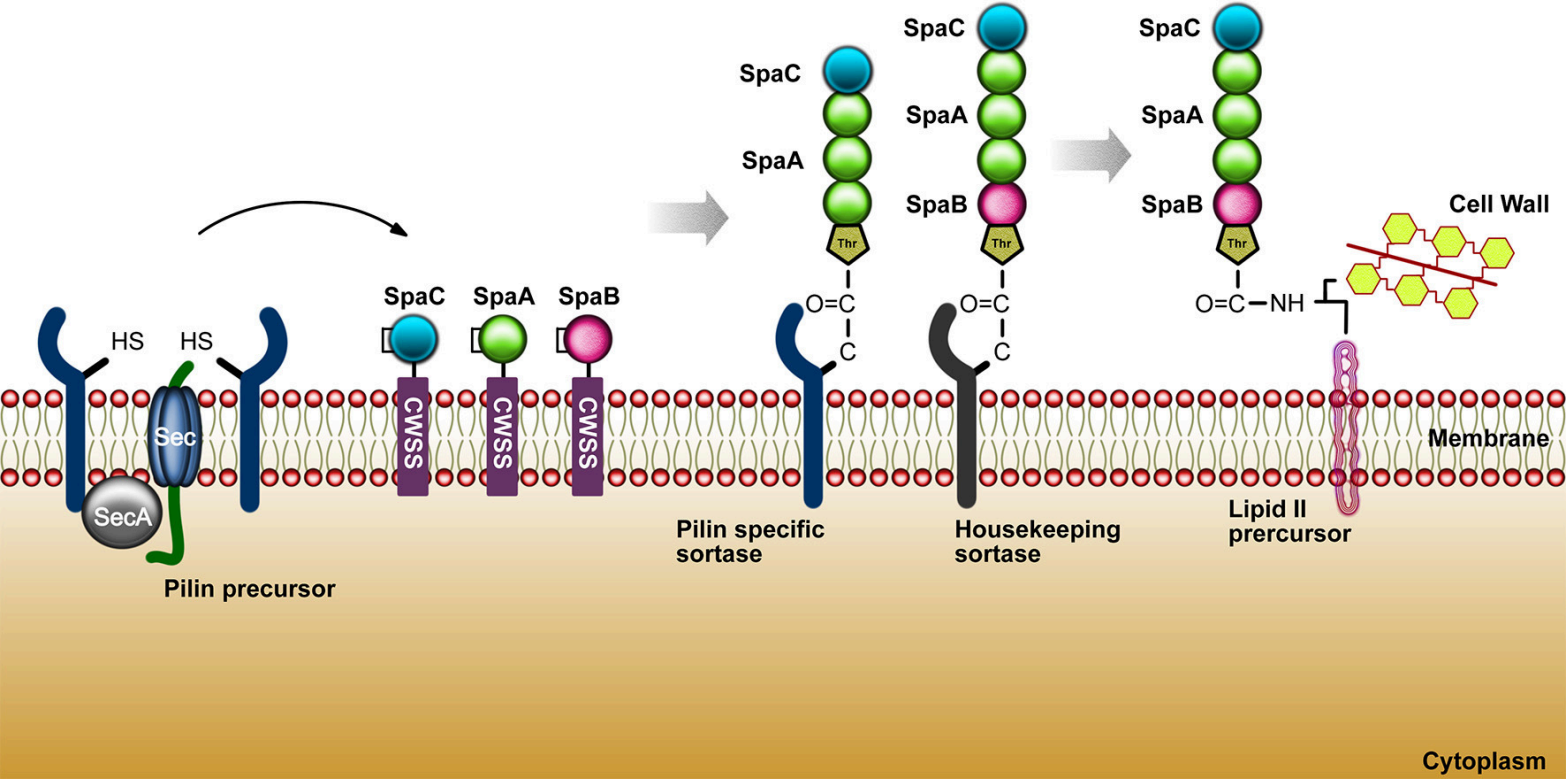
The sortase mechanism in gram-positive bacteria. As shown above for *C. diphtheriae*, pilins are thought to be translocated upon the cytoplasmic membrane (Export) and hold within the secretory pathway by the cell wall sorting signal CWSS (Retention). These biomolecules (cysteine transpeptidases) join proteins conducting an appropriate sorting signal to strategically positioned amino groups on the cell surface.

The bacterial adhesion process to the host cells was firstly elucidated through research with *E. coli* in 1908, in which it was shown that these bacteria, via multimeric pili, could hemagglutinate animal cells. In order to better understand important features associated to bacterial adhesion, some studies has been focusing on specific proteins as the pili, monomeric adhesins and bacterial tissue tropism ability. Research intended to explore the different manners by which bacteria may adhere to their host and how the host respond to it has opened a big field of study. Nowadays, it is known that pathogenic and commensal bacteria are able to express molecules that can help their interaction with eukaryotic host cells. Under this process, the underlying mechanism may be either beneficial for bacterial colonization or disadvantageous when the adhesion activates specific processes that may eliminate bacteria from the environment through phagocytosis and immune cell infiltration (Kline et al., 2009). The species from the genus *Corynebacterium* have in common a rich variety of lipoglycans, such as lipoarabinomannans (LAM) and lipomannas (LM) (Brennan and Nikaido, 1995). *C. diphtheriae* presents a lipoglycan surface component called CdiLAM, a novel LAM variant that contributes to adhesion through an interaction with human respiratory epithelial HEp-2 cells (Moreira et al., 2008). Another adhesive component in *C. diphtheriae*, identified as surface-associated protein DIP1281 and annotated as hypothetical invasion-associated protein, was investigated by atomic force microscopy and fluorescence staining. The DIP1281 gene product seems to be related to the organization of the outer protein layer of *C. diphtheria* (Ott et al., 2010). The bacterial adhesins may recognize and colonize through of a differential regulation of genes either by the activation of structures such as hair-like, characteristic named pili or fimbriae, or identified as non-pilus adhesin, when are related with microbial cell surface (Soto and Hultgren, 1999). Gram-positive bacteria possess several surface proteins anchored to the cell wall through a sortase-dependent mechanism. The class II of sortase (minor class, formed by sortases with N-terminal signal peptide and a C-terminal membrane anchor) are the most frequent in corynebacteria, streptococci, and enterococci (Cossart and Jonquières, 2000; Ton-That et al., 2004). The first description of the pili molecule in gram positive bacteria, dates to around 1967 with a study of 45 *Corynebacterium renale* strains. The fibrils identified presented a great similarity to pili protein structures, previously described in gram negative bacteria. Using electron microscopy, the pilus characteristics of *C. renale* were observed based in morphological analyses of quantity and length (Yanagawa et al., 1968). According to Trost et al. (2010), there are gene clusters encoding sortase genes related to the adhesive pili synthesis from *C. pseudotuberculosis* FCR41. The pili structures are comprised of the major pilin, SpaA and SpaD; minor pilin, SpaB and SpaE; and tip pilin, SpaC, SpaF. A complete pilus structure or even the minor pilins may perform an initial contact to the host cell receptors facilitating the delivery of virulence factors. *C. pseudotuberculosis* FCR41 presents a housekeeping sortase (*srtD*), responsible for the pili anchorage to the cell wall and located in another region of the genome (Trost et al., 2010). In the research of Ton-That and Schneewind (2003), it was observed that *C. diphtheriae* strain NCTC13129 also shows pili regions. This work showed three pili clusters: SpaA, major pilin protein; SpaB, minor pilin; and, SpaC, as a pilus tip protein. The housekeeping gene of *C. diphtheriae* is *srtF*, localized elsewhere on the chromosome (Ton-That and Schneewind 2003). In 2006, a second and third pilus were characterized, in which the second pilus cluster was identified as presenting a major pilin protein (SpaD) and minor pilus protein (SpaE and SpaF), and the third pilus cluster contains a major pilin (SpaH) and two minor pili proteins (SpaG and SpaI) (Gaspar and Ton-That, 2006). Moreover, the length of the filament is directly proportional to the abundance of the SpaH pilus (Swierczynski and Ton-That, 2006). In 2011, Trost et al. worked with two *C. ulcerans strains* to search for candidate virulence factors. The proteomes of *C. ulcerans* 809 and *C. ulcerans* BR-AD22 were analyzed and they found twelve virulence factors (*cpp, pld, spaF, spaE, spaD, spaC, spaB, rpfl, cwIH, nanH, vps1*, and *tspA*) commonly shared by both strains, and two exclusive to *C. ulcerans* 809 (*rbp* and *vsp2)*. Both *C. ulcerans* strains present the genes *srtB* and *srtC* in the *spaDEF* region. Moreover, a second pilus gene cluster was observed, the *spaBC, in* which *spaB* gene is responsible for minor pilus protein coding and *spaC* codes a tip protein (Trost et al., 2011). Figure 3 summarizes the sortase mechanism in gram-positive bacteria.

In addition to these molecules associated to anchorage to the cell wall, the bacteria have shown a system of others molecules able to control a large number of physiological functions including sporulation, competence, antibiotic resistance, and the transition to stationary phase (Mitrophanov and Groisman, 2008), called two-component systems (TCS). These systems are also able to mediates the adaptive responses to the environment (Koretke et al., 2000), including the pathogen virulence ability (Pérez et al., 2001).

Furthermore, some studies have described the characterization of virulence factors of *Corynebacterium* species by means of host model, such as *Caenorhabditis elegans*. Studying 42 isolates of *C. diphtheriae*, Broadway et al. showed that most of them expressed the heterotrimeric SpaA pili, characterizing the bacterial adherence to pharyngeal epithelial cells. In addition, they observed that both non-toxigenic and toxigenic strain of *C. diphtheriae* (lack pili) aggravated the death of the nematode comparing with the control NCTC13129, toxigenic and piliated strain (Broadway et al., 2013). Recently, a work using the same model, investigated other characteristics related with the pathogenicity of *C. diphtheriae, C.ulcerans, and C. glutamicum*, in addition of pili and nematode killing analysis (Antunes et al., 2017).

Another element associated as virulence factor is the *phoP* gene, that takes part in the PhoPR two-component system regulation, in which Tiwari et al. analyzed the role of the PhoP protein from *C. pseudotuberculosis* Cp1002. Curiously, the mutant Cp1002 strain produced with absence to phoP gene, showed a reduction of cell adhesion rates that affects the surface properties (Tiwari et al., 2014).

### Toxicity of corynebacteria

**Figure 4:**
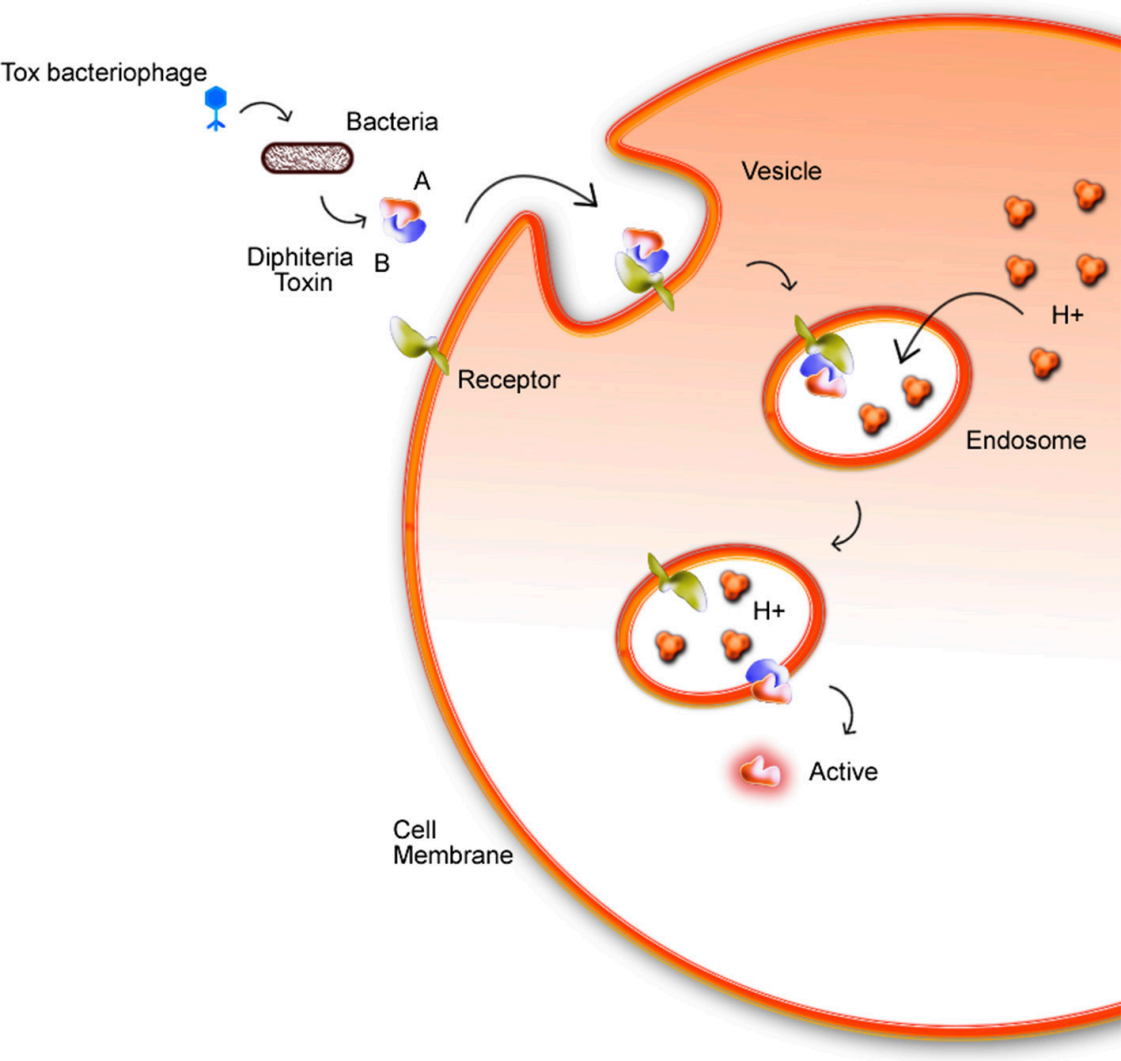
Mechanism in which the bacterial DT invades the host cell membrane. The toxin enters through the membrane by endocytosis with the aid of fragment B. Fragment A is then actively exposed to the cytoplasm. In acidic endosomes, its translocation domain inserts into endosomal membranes and facilitates the transport of the catalytic domain (DTA) from endosomal lumen into the host cell cytosol. Here, DTA ADP-ribosylates elongation factor 2 inhibits protein synthesis and leads to cell death (Schnell et al., 2016).

Phages are widely found in the environment, such as soil, and they present a multiple cycles repeated daily by cellular lysis. The presence of phages in corynebacteria is described since 1970s (Ogata et al., 1980; Annie et al., 1987). Freeman's studies identified that some *C. diphtheriae* strains, using a mechanism of phage-mediated conversion, could produce the diphtherial toxin (Freeman and Morse, 1951; Hard, 1975). This discovery caught the attention for future works about corynebacteriophages, such as one of the first described comparative analysis of tox^+^ and tox^−^performed by Randall and Lane (Holmes and Barksdale, 1970). The *tox* gene is found in corynebacteriophage regions: β tox^+^, γ tox^+^, and ω tox^+^, inside the chromosomes of *C. diphtheriae, C. ulcerans*, and *C. pseudotuberculosis*, but in this last one just in the biovar equi. Chromosomal genes interfere directly in Diphtheria Toxin (DT) expression and may cause, for example, an increase of DT production resulting from a low intracellular amount of iron due to inhibition of the regulatory *dtx*R gene (Guaraldi et al., 2014). *C. diphtheriae* strains produce DT, which is responsible for inhibiting protein synthesis in eukaryotic cells. DT is composed of two subunits, A and B fragments. DT binds to a specific receptor via B fragment while the A fragment, that is the active region, is translocated by an endosome into the host cell cytoplasm (Figure 4). The *tox*^+^ phage may convert non-toxinogenic *C. diphtheriae, C. ulcerans*, and *C. pseudotuberculosis* to toxinogenic bacteria (Buck et al., 1985; Naglich et al., 1992). In a recent work (Schnell et al., 2016), diphtheria toxin (DT, 58 kDa), which is the causative agent of diphtheria is efficiently taken up into human cells and its catalytic domain (DTA, 21 kDa) acts as an extremely potent enzyme in the cytosol. Histidines residues (diphthamide) are modified by DTA covalently transfers, inhibiting protein synthesis and causing cell death.

Sing et al. demonstrated in 2003 that there are differences between the *tox* genes from *C. ulcerans* and *C. diphtheriae* strains. The DT from both species are about 95% identical in nucleotide and amino acid sequences, where the differences corresponding around to one and three amino acids located in the signal and catalytic region of the A fragment, respectively and 23 amino acids of DT are different between *C. diphtheriae* and *C. ulcerans* in B fragment (Sing et al., 2003).

Another toxin related to species from genus *Corynebacterium* is phospholipase D (PLD). This toxin, which is encoded by the *pld* gene, has been indicated as the leading virulence factor for this bacteria. Furthermore, studies suggest that PLD activity facilitates the dissemination and infiltration process into host tissues (Hodgson et al., 1992). Through pan-genomic analyses, Soares et al. identified the presence of *pld* in 14 of 15 *C. pseudotuberculosis* strains. One of these strains, *C. pseudotuberculosis* 31, presents a frameshift mutation near the 3'-end of the *pld* gene that could be implicated with a minor ability of this strain to interact with the host. However, from all strains, *C. pseudotuberculosis* strain 31 was the only one in this study to harbor the *tox* gene (Soares et al., 2013). In *C. ulcerans* there is an extracellular neuraminidase (NanH), a potential virulence factor. This same NanH also occurs in *C. diphtheriae, C. pyogenes, C. pseudotuberculosis*, and *C. belfanti* (Trost et al., 2010). The two *C. ulcerans* studied by Trost et al. also presented PLD (Trost et al., 2011).

### Survival inside the macrophage

Inside macrophages, bacteria are exposed to a broad range of different environmental stresses that may affect the metabolism, interfere with the structure and, consequently, the function of macromolecules. The intracellular medium may be altered by a nitrosative surface, oxidative/thermal osmotic shock, and starvation stresses that will trigger survival mechanism of the bacteria (Kazmierczak et al., 2005).

*C. pseudotuberculosis*, the causative agent of caseous lymphadenitis (CL), presents a lipidic surface associated with pathogenicity and virulence. In a study with this lipidic surface, it was demonstrated that the toxicity observed in extracted lipid may cause a high susceptibility to mouse peritoneal macrophages, due to the necrotizing action of the *C. pseudotuberculosis* surface lipids, whereas this same cytotoxic effect was not observed in macrophages from rabbits and guinea pigs (Hard, 1975).

In order to understand how *C. pseudotuberculosis* 1002 is able to survive in the environment to infect the host, Pinto et al. analyzed it through transcriptional profile with regards to acid osmotic and thermal shock stresses. The most relevantly expressed genes were related to the adhesion process, stress response, and oxidoreduction, where the genes involved in virulence ability were affected. In the adhesion process, the Cp1002_0988 gene, one of the three genes comprising this mechanism was very relevant under osmotic stress because it was located within one of the pathogenicity islands predicted for *C. pseudotuberculosis* 1002. Besides that, this gene presented 6.8-fold change in expression levels in comparison with the control, whereas the other two genes, Cp1002_1764 and Cp1002_1765 presented 2.7- and 4-fold changes in expression. Regarding the genes that constituted the cellular oxireduction processes, they observed that some genes showed functions related to bacterial persistence in this environment. Curiously, some genes are involved in both adhesion and oxidoreduction and were fundamentally important due to the relevance in virulence (Pinto et al., 2014).

In addition, a *phoP* gene study was performed to analyze the adhesion importance and invasion in a wild type and a *phoP* mutant strain of *C. pseudotuberculosis* 1002. There was a change in genes possibly involved in the metabolism of lipids composing the cell envelope that may affect the *phoP* mutant interaction with the macrophages (Tiwari et al., 2014).

In analysis of osmostic stress response of *C. glutamicum*, it was demonstrated that the bacteria might adapt its metabolism according to the adverse conditions, meaning the osmolarity may only affect the cells if a variation in this coefficient exists. Besides that, the production of lysine increased in low dilution rates and with glucose consumption (Varela et al., 2004). Regarding the oxidative stress, carbon fluxes in the central metabolism of *C. glutamicum* were affected under deletion of the regulatory protein McbR, redirecting from the pentose phosphate pathway into glycolysis in response to the deletion. Furthermore, the deletion resulted in the increasing of pyruvate dehydrogenase (Krömer et al., 2008).

The activity and regulation of a zinc uptake regulated (Zur), in *C. diphtheriae*, Smith et al. identified three genes whose transcription is repressed by Zur: *cmrA, zrg*, and *troA*. Besides, they showed that *zur* gene transcription is made by a promoter, and it one is regulated by *zur* gene product, being its product modulate by zinc presence in the environment, also indicating the ability of *C. diphtheriae* to integrate different signals from a variety of metals to survive (Smith et al., 2009).

### Host-pathogen relationship in the infection by *C. pseudotuberculosis* and immune response

As a major *C. pseudotuberculosis* virulence factor, the exotoxin pld has already been described as an important molecule in the host-pathogen relationship, since it is implicated in the dissemination of the bacteria from the original area of the infection to other organs, through the hydrolysis of sphingomyelin from the host's tissues and cell membranes (Pépin et al., 1989). The pld protein is seen to be associated with infection although is described with a small role but it seen important for the viability after the infection (McKean et al., 2007), and recently it was described that lymphocyte activation is inhibited by the *C. pseudotuberculosis* sphingomyelinase through the suppression of the ORAI receptors (Combs and Lu, 2015). The importance of the pld exotoxin in the host-pathogen relationship can be understood by the observation that anti-pld antibodies can inhibit the bacterial dissemination in the host and is related to the protection induced by vaccination (De Rose et al., 2002; Moura-Costa et al., 2008). The endo-β-N-acetylglucosaminidase CP40 is a secreted molecule that have been recently described as an important *C. pseudotuberculosis* virulence factor (Shadnezhad et al., 2016) and the vaccination of mice with this protein showed high protection rates (Droppa-Almeida et al., 2016). Other molecules that are involved in the establishment of the infection and to its dissemination to other organs are the lipids that are found in the bacterial wall, since the amount of these lipids in different bacterial strains were closely related to the virulence presented by each isolate (Muckle and Gyles, 1983).

Since *C. pseudotuberculosis* is a facultative intracellular pathogen, the protective immune response to the bacterium is based on a Th1 cellular response, and the infection with virulent strains of the bacteria induces the production of Th1 response-related cytokines such as IFN-gamma (Meyer et al., 2005; Vale et al., 2016). Indeed, it was seen that the production of IFN-gamma and TNF following the stimulation of murine splenocytes with the bacterium is driven by the activation of MAPK p38 and ERK 1/2 signaling pathways (de Souza et al., 2014). Mice inoculated with anti-IFN-gamma antibodies showed an increased bacterial proliferation in the organs (Lan et al., 1998), but this work also shown that the innate immune response is also important, since the inoculation of anti-TNF antibodies also led to a higher bacteremia. It has also been observed that *C. pseudotuberculosis*-infected small ruminant' peripheral mononuclear blood cells are capable to produce significant amounts of IFN-gamma when stimulated *in vitro* with bacterial antigens, and goats have a more intense production of this cytokine than sheep (Rebouças et al., 2011). This situation can be related to the fact that caseous lymphadenitis lesions in sheep are more disseminated and can be found in organs like spleen, lungs, and liver, and in goats these lesions are more likely to be found in superficial lymph nodes (Moura-Costa et al., 2008; Bastos et al., 2011).

The humoral immune response is also activated in the infection by *C. pseudotuberculosis*. In a murine model, it was observed that there is a peak in the production of IgG1 and IgG2a 60 days after an experimental infection with a pathogenic strain of the bacteria (Vale et al., 2016). Recent experiments on the development of vaccines against the bacteria showed that the production of IgG2a is correlated with a higher protection against a virulent challenge in mice that were previously inoculated with *C. pseudotuberculosis* recombinant proteins (Silva et al., 2014; Brum et al., 2017). Bastos et al. (2011-2013) observed that sheep that produced higher amounts of specific IgM in the acute phase of the disease present a better prognostic in the clinical progression of caseous lymphadenitis and are more likely to develop asymptomatic infections. Among the several bacterial antigens that induces the production of specific antibodies, the exotoxin pld is considered a major immunodominant antigen, and it was observed that 100% of goats that were infected with the bacteria present specific IgG against pld, but only 70% of the infected sheep present this same pattern of antibody production (Hoelzle et al., 2013). Anti-pld antibodies are considered good biomarkers in the serodiagnosis of the infection in small ruminants, and satisfactory specificity and sensitivity levels were seen when recombinant pld or bacterial culture supernatants were employed as antigens in the development of immunodiagnosis assays (Sutherland et al., 1987; Menzies et al., 2004; Rebouças et al., 2011).

### Mechanisms of antibiotic resistance in *Corynebacterium*

Studies point out that different species can show antibiotic resistance to different chemical compounds. Additionally, these results could to explain that extrachromosomal genetic elements are associated in the transmission of resistance genes in both pathogenic and potentially pathogenic (Adderson et al., 2008; Fernandez-Roblas et al., 2009). Usually, in species of *Corynebacterium*, the antibiotic resistance genes are located on large plasmids, e.g., resistance to tetracycline, chloramphenicol, erythromycin, and streptomycin on plasmid pTP10 described in *C. xerosis*. Others elements as the fluoroquinolones are described associated to the mechanism of resistance, also the tetracyclines are observed in phenotypic studies being found in 97% of the tested strains. Some glycopeptides can be described as component of the antibiotic resistance such as the vancomycin, an antibiotic recommended by many authors in the empirical treatment of invasive infections.

On the basis of phenotypic and genotypic studies is possible to understand the mechanism of antibiotic resistance that are proper to each species of the genus *Corynebacterium* associated with infections. These same studies could improve the knowledge about the sensitivity to antibiotics of different multidrug-resistant species of the genus Corynebacterium. Currently, some candidates are indicated as highest efficacy in treatment of infections such as tigecyclin, chinupristin/dalphopristin, glycopeptides, daptomycin, and linezolid (Olender, 2012).

## Main features of non-pathogenic species

### Biotechnology applications

Microorganisms produce several things that are useful for mankind and that is the reason behind importance of these tiny creature (Demain, 1990). These material could be explained as small molecules, usually described as metabolites that are broadly explore to vegetative growing (primary) and those that are described as inessential (secondary) or large molecules explained as nucleic acids, proteins, carbohydrates). For centuries, these organisms have been utilized for the production of daily goods such as beer, bread, and wine. For example, filamentous fungi have been extensively used for the commercial production of organic acids, with, 1 billion pounds of citric acid with a market value of US$1.4 billion produced annually. The Embden-Meyerhof pathway's product followed by the tricarboxylic acid cycle for the production of citric acid; the process is controlled by the inhibition of phosphofructokinase by citric acid. *Aspergillus niger* is used in iron and manganese deficient media for commercial processes. High level citric acid production is proportional to high intracellular concentration of fructose 2,6-biphosphate, a potential glycolysis activator (Demain, 2000).

At present, the net annual global expenditure of amino acids production is valued to be more than 2 million tons. The yearly demand for amino acids like MSG-based flavor enhancers or food additives including D,l-methionine, l-lysine, and l-threonine is around to be considerably higher than 1 million tons each. It is estimated that the pharmaceutical industry uses, mainly for intravenous and enteral nutrition, demand a value per year around 15,000 tons of amino acids (Kusumoto, 2001).

The genomic structure and metabolism of *C. glutamicum* are well characterized making this a microorganism better targeted for biotechnological utilization among this genus. This organism is frequently employed to production of L-amino acids (Becker and Wittmann, 2012b). However, some works already described this microorganism can be engineered for production of several “first-class” chemicals like succinate (Litsanov et al., 2012) or isobutanol (Blombach et al., 2011). Regarding these l-amino acids, the most relevant are l-glutamate and l-lysine based on production scales (Becker and Wittmann, 2012a). Furthermore, there are the expectation to create qualified producers for others amino acids such as l-leucine, l-serine, and l-methionine, which are uphold by a detailed knowledge into the corresponding regulation and their amino acid biosynthetic pathways in *C. glutamicum*.

### Fermentation

The fermentation process can create almost 1,200 million pounds of monosodium glutamate per year, using different species of the genera *Corynebacterium* (e.g., *C. glutamicum*) and *Brevibacterium* (e.g., *B. flavum* and *B. lactofermentum*) (Demain, 2000). Additionally, these microorganisms can be utilized for the amino acid production, for example the l-glutamic acid, which is a commercially very important, frequently used as a food preservative in order to flavor enhancer, such as sodium salt (Shyamkumar et al., 2014). In addition, another one amino acid, with strong commercial importance, is the l-lysine that also is produced by batch fermentation. Usually, the industrial process looks for efficient approaches with short cost and time, using immobilized cells for aerobic processes or free cells. However, such type of applicability is still defiant. A current work (Razak and Viswanath, 2015) trying figuring out the better way to ascertain whether free cells or immobilized cells were useful for l-lysine production, showed that immobilized cells of the strain *C. glutamicum* MH 20-22 B, were more prominent as they yield more l-lysine compared to free cells.

## Important biochemical pathways

### Lysine biosynthesis pathway

The amino acid L-lysine is produced by fermentative processes, conducted by bacteria can be produced together with methionine, threonine, and isoleucine by a branched of biochemical pathways. This pathway is harshly controlled for *E. coli* species, which includes a set of kinases proteins that are able to regulate its production. Additionally, when a large amount of the final product is abundant the initial enzyme is inhibited, in which no overproduction occurs.

Nevertheless, in some lysine-fermentation organisms (e.g., several mutants of *C. glutamicum* and its relatives), the only protein kinase able to act in the regulation is the aspartate kinase, which its presence is regulated via coordinated feedback inhibition by threonine and lysine. Curiously, some strains of *C. glutamicum* cannot to grow up unless methionine and threonine are present in the medium, its due to some of these strains are absent to the homoserine dehydrogenase. This observation was described that while the threonine supplement is maintained at a limiting concentration, the intracellular concentration of threonine will be used as the limiting factor and feedback inhibition of aspartate kinase will be bypassed (Demain, 2000). On the other hand, recently Pérez-García et al. (2016) reported the modification in phosphotransferase system (PTS) in *C. glutamicum strain GRLys1* produced l-lysine and l-pipecolic acid quickly. That can also be an alternative way for the production of l-lysine through *C. glutamicum* (Pérez-García et al., 2016).

### Carotenoids biosynthesis pathway

The literature describe the carotenoids are yellow to red colored pigments originating from the terpenoid biosynthetic pathway. They are very abundant in plants and microorganisms, where they can have diverse functions such as photo protection or light harvesting molecules or as membrane stabilizers (Lee and Schmidt-Dannert, 2003; Sandmann and Yukawa, 2005). Carotenoids are derived from the universal precursor isopentenyl pyrophosphate (IPP) and its isomer dimethylallyl pyrophosphate (DMPP) (Rodríguez-Villalón et al., 2008). Moreover, IPP and DMAPP can influence the yield and rates of isoprenoid production by its enhancing of cellular metabolic flux, using them as strategy to the improvement of these molecules (Martin et al., 2003; Leonard et al., 2010).

The C50-terpene decaprenoxanthin and its glucosides are described as the predominant carotenoids in *C. glutamicum* (Krubasik et al., 2001a). Currently, just three different C50 carotenoid biosynthetic pathways have been described: the biosynthetic pathways of the ε-cyclic C50 carotenoid decaprenoxanthin in *C. glutamicum* (Krubasik and Sandmann, 2000; Krubasik et al., 2001b), the β-cyclic C50 carotenoid C.p. 450 in Dietzia sp. CQ4 (Tao et al., 2007) and the γ-cyclic C50 carotenoid sarcinaxanthin in *Micrococcus luteus* NCTC2665 (Netzer et al., 2010). In addition, only five species of Corynebacterium genus have been identified to include carotenoid pigments i.e., *C. michiganense* (Saperstein and Starr, 1954), *C. erythrogenes* (Hodgkiss et al., 1954), *C. fascians* (Prebble, 1968), and *C. poinsettiae* (Starr and Saperstein, 1953). *C. poinsettiae* (*Curtobacterium flaccumfaciens*) e.g., is known to produce the C50 carotenoids bacterioruberin, bisanhydrobacterioruberin, and C.p. 450 (Sandmann and Yukawa, 2005).

As described in some works, *C. glutamicum* possesses a carotenogenic gene cluster, observed based on transposon mutant analysis and biochemical evidence, that encodes responsible enzymes for the entire decaprenoxanthin biosynthesis starting from DMPP (Krubasik and Sandmann, 2000; Krubasik et al., 2001a). The farnesyl pyrophosphate (FPP, C15) and geranylgeranyl pyrophosphate (GGPP, C20) are suggested to be the promptly pioneers of the C30 and C40 carotenoids biosynthesis are generated from DMPP by prenyl transferase CrtE (Krubasik and Sandmann, 2000). Subsequently, the colorless carotenoid phytoene is produced by phytoenesynthase (CrtB) activity. Finally, the red-colored lycopene is produced by four subsequent desaturation reactions by phytoene desaturase (CrtI) (Krubasik and Sandmann, 2000; Krubasik et al., 2001b). The crtEb gene that produce the lycopene elongase enzyme is able to catalyze the reaction of the elongation of lyconpene to the acyclic C50 carotenoid flavuxanthin. Posteriorly, the reaction of flavuxanthin to decaprenoxanthin is catalyzed by heterodimeric carotenoid-ε-cyclase, encoded by crtYf and crtYe genes (Krubasik et al., 2001b; Eggeling and Bott, 2005; Netzer et al., 2010). Despite the mono/diglucosylated decaprenoxanthin can be explored in *C. glutamicum*, the genes and enzymes for glucosylation of decaprenoxanthin are still unidentified (Netzer et al., 2010).

### Histidine biosynthesis pathway

The first genetic studies on histidine biosynthesis were targeted using the *C. glutamicum* AS019, which was derivated of *C. glutamicum* ATCC 13059. The genes hisA, that is able to codify the 1-(5-phosphoribosyl)-5-[(5phosphoribosylamino)methylideneamino]imidazole-4carboxamide (5′ProFAR) isomerase, and the gene hisF encoding one subunit of the imidazole glycerol phosphate synthase, were analyzed by aggregation of analogous histidine auxotrophic *E. coli* mutants (Jung et al., 1998). The gene hisH, that is able to codify the second subunit of imidazole glycerol phosphate synthase (Kim and Lee, 2001), and the genes hisG and hisE, to the ATP phosphoribosyltransferase and phosphoribosyl-ATP pyrophosphatase, were respectively analyzed using the same methodology (Kwon et al., 2000). With the access to complete genome sequence of *C. glutamicum* (strain ATCC 13031) (Ikeda and Nakagawa, 2003; Kalinowski et al., 2003), several metabolic pathways were able to be reconstructed and better understood, including histidine biosynthesis. Nine of the 10 enzymatic activities needed for histidine biosynthesis were identified by genome annotation, these were the genes hisAEFGH, already known from *C. glutamicum* AS019, the genes *hisI*, encoding phosphoribosyl-AMP cyclohydrolase, *hisB*, coding for imidazoleglycerol-phosphate dehydratase, *hisC*, coding for histidinol-phosphate aminotransferase, and *hisD*, encoding histidinol dehydrogenase, which catalyzes the final two steps of histidine biosynthesis in *C. glutamicum*.

It been more than three decades that we are introduced with modern biotechnology. With the birth of recombinant DNA technology in 1972, biotechnology has been driven to new heights that headed toward the constitution of a new industry. Along with recombinant DNA technology, new microbial biotechnology including fermentation, high-throughput screening for drug target and novel metabolites, microbial physiology, and strain improvement, bioreactor design and downstream processing, cell fusion, cell immobilization, protein engineering, metabolic engineering, and evolutionary characterization of enzymes (Wendisch et al., 2016).

The genomics, transcriptomics, proteomics considered as application of high-throughput technologies have been used in the development of *C. glutamicum* strains by the use of the biotechnology industry. These new technologies helped immensely in the strain development production of *C. glutamicum*. For example, the genome-reduced of *C. glutamicum* strain MB001 was biotechnologically modeled for the production of the decaprenoxanthin, carotenoids lycopene, and astaxanthin (Heider et al., 2014), and the amino acids L-citrulline (Eberhardt et al., 2014) (Eberhardt et al., 2014). Correspondingly, MB001-based strains were developed for the production of the amino acids L-arginine, L-ornithine, L-proline, and the diamine putrescine (Jensen et al., 2015). In recent times, CRISPR interference (CRISPRi) technology using the inactive Cas9 protein was performed on L-lysine and L-glutamate production to determine the effects of target gene repression (Cleto et al., 2016). Effects on amino acids produced by CRISPRi/dCas9-mediated target gene repression were comparable to levels achieved by target gene deletion. For many target genes, this robust methodology may potentially be operated in parallel. The use of signal peptide is one of the approach identified for enhancing secretion efficiency. However, when compared with *E. coli, C. glutamicum* has some disadvantages, such as lower efficiency of transformation and a low number of available expression systems. To achieve and overcome such challenges, it is needed to the development of extraordinary approaches for expression and secretion systems that leads to gain a deeper understanding and increase production workhorse in the field of microbial biotechnology (Lee et al., 2016; Wendisch et al., 2016).

## Conclusion

We conclude that the proposed work adds significantly information in the medical, veterinary, and biotechnology area to *Corynebacterium* genus and its species. Additionally, the review explores general important contributions such as the cell division and Genomic/Transcriptomic. Also, specific details to pathogenic for example highlighting the virulence factors and curiosities about the host-pathogen relationship, an infamous context extremely important to be discussed to these species and non-pathogenic species, such as the amino acid production strongly important to the food production.

## Author contributions

AO—Writing and reading of review; LO, LB, SJ, ST, FA, and RP—writing of specific parts of manuscript; HF, AS, and PG—Support in the manuscript development; RW, and VA—Leadership and supervision of work.

### Conflict of interest statement

The authors declare that the research was conducted in the absence of any commercial or financial relationships that could be construed as a potential conflict of interest.

## Supplementary material

Supplementary Table1 Detailed list of corynebacterium genomes.

## References

[B1] Abdolrasouli A. Roushan A. (2013). Corynebacterium propinquum associated with acute, nongonococcal urethritis. Sex. Transm. Dis. 40, 829–831. 10.1097/OLQ.0000000000000027 24275738

[B2] Adderson E. E. Boudreaux J. W. Hayden R. T. (2008). Infections caused by coryneform bacteria in pediatric oncology patients. Pediatr. Infect. 27, 136–141. 10.1097/INF.0b013e31814fab12 18174873

[B3] Albersmeier A. Pfeifer-Sancar K. Rückert C. Kalinowski J. (2017). Genome-wide determination of transcription start sites reveals new insights into promoter structures in the actinomycete *Corynebacterium glutamicum*. J. Biotechnol. 257, 99–109. 10.1016/j.jbiotec.2017.04.008 28412515

[B4] Al-Dilaimi A. Albersmeier A. Kalinowski J. Rückert C. (2014). Complete genome sequence of *Corynebacterium vitaeruminis* DSM 20294T, isolated from the cow rumen as a vitamin B producer. J. Biotechnol. 189, 70–71. 10.1016/j.jbiotec.2014.08.036 25193714

[B5] Alfo J. Lindblad-toh K. (2013). Comparative genomics as a tool to understand evolution and disease. Genome Res. 23, 1063–1068. 10.1101/gr.157503.113.Freely 23817047 PMC3698499

[B6] Alvarez H. M. Pucci O. H. Steinbüchel A. (1997). Lipid storage compounds in marine bacteria. Appl. Microbiol. Biotechnol. 47, 132–139.

[B7] Ang L. M. Brown H. (2007). *Corynebacterium accolens* isolated from breast abscess: possible association with granulomatous mastitis. J. Clin. Microbiol. 45, 1666–1668. 10.1128/JCM.02160-06 17344355 PMC1865888

[B8] Annie T. Carlos B. Bonnassie S. (1987). Characterization of the corynebacteriophage CG33. J. Gen. Microbiol. 133, 2945–2922. 3449603 10.1099/00221287-133-10-2945

[B9] Ansorge W. J. (2009). Next-generation DNA sequencing techniques new biotechnology. N. Biotechnol. 25, 195–203. 10.1016/j.nbt.2008.12.009 19429539

[B10] Antunes C. A. Clark L. Hacker E. Ott L. Simpson-louredo L. De Luna G.. (2017). *Caenorhabditis elegans* Star formation and negative chemotaxis induced by infection with corynebacteria. Microbiology 162, 84–93. 10.1099/mic.0.000201. 26490043

[B11] Antunes J. M. Ribeiro M. G. L. Castro Demoner Ramos J. N. Pereira Baio P. V. Simpson-Louredo L. Santos C. S.. (2015). Cutaneous abscess caused by *Corynebacterium lactis* in a companion dog. Vet. Microbiol. 178, 163–166. 10.1016/j.vetmic.2015.04.014 25937144

[B12] Auzias A. Bollet C. Ayari R. Drancourt M. Raoult D. (2003). *Corynebacterium freneyi* bacteremia. J. Clin. Microbiol. 41, 2777–2778. 10.1128/JCM.41.6.2777-2778.2003 12791929 PMC156481

[B13] Baird G. J. Fontaine M. C. (2007). *Corynebacterium pseudotuberculosis* and its role in ovine *Caseous lymphadenitis*. J. Comp. Pathol. 137, 179–210. 10.1016/j.jcpa.2007.07.002 17826790

[B14] Bastos B. L. Guimarães J. E. Ayres M. C. Guedes M. T. Moura-Costa L. F. de Burghgrave U. S.. (2011). Haptoglobin and fibrinogen concentrations and leukocyte counts in the clinical investigation of *Caseous lymphadenitis* in sheep. Vet. Clin. Pathol. 40, 496–503. 10.1111/j.1939-165X.2011.00355.x 22092668

[B15] Becker J. Wittmann C. (2012a). Bio-based production of chemicals, materials and fuels - *Corynebacterium glutamicum* as versatile cell factory. Curr. Opin. Biotechnol. 23, 631–640. 10.1016/j.copbio.2011.11.012 22138494

[B16] Becker J. Wittmann C. (2012b). Systems and synthetic metabolic engineering for amino acid production - the heartbeat of industrial strain development. Curr. Opin. Biotechnol. 23, 718–726. 10.1016/j.copbio.2011.12.025 22244788

[B17] Bernard K. (2005). Corynebacterium species and coryneforms: an update on taxonomy and diseases attributed to these taxa. Clin. Microbiol. Newsl. 27, 9–18. 10.1016/j.clinmicnews.2005.01.002

[B18] Bernard K. (2012). The genus Corynebacterium and other medically relevant coryneform-like bacteria. J. Clin. Microbiol. 50, 3152–3158. 10.1128/JCM.00796-12 22837327 PMC3457441

[B19] Blombach B. Riester T. Wieschalka S. Ziert C. Youn J. W. Wendisch V. F.. (2011). *Corynebacterium glutamicum* tailored for efficient isobutanol production. Appl. Environ. Microbiol. 77, 3300–3310. 10.1128/AEM.02972-10 21441331 PMC3126470

[B20] Brennan P. J. Nikaido H. (1995). The envelope of mycobacteria. Annu. Rev. Biochem. 64, 29–63. 10.1146/annurev.bi.64.070195.000333 7574484

[B21] Broadway M. M. Rogers A. E. Chang C. Huang I.H. Dwivedi P. Yildirim S. (2013). Pilus gene pool variation and the virulence of *Corynebacterium diphtheriae* clinical isolates during infection of a nematode. J. Bacteriol. 195, 3774–3783. 10.1128/JB.00500-13 23772071 PMC3754579

[B22] Brum A. A. de Fatima Silva Rezende A. Brilhante F. S. Collares T. Begnine K. Seixas F. K. Collares T. V.. (2017). Recombinant esterase from *Corynebacterium pseudotuberculosis* in DNA and subunit recombinant vaccines partially protects mice against challenge. J. Med. Microbiol. 66, 635–642. 10.1099/jmm.0.000477 28516859

[B23] Buck G. A. Cross R. E. Wong T. P. Loera J. Groman N. (1985). DNA relationships among some tox-bearing corynebacteriophages. Infect. Immun. 49, 679–684. 2993167 10.1128/iai.49.3.679-684.1985 PMC261242

[B24] Burr H. N. Wolf R. F. Lipman N. S. (2012). *Corynebacterium bovis*: epizootiologic features and environmental contamination in an enzootically infected rodent room. J. Am. Assoc. Lab. Anim. Sci. 51, 189–198. 22776119 PMC3314522

[B25] Camberg J. L. Hoskins J. R. Wickner S. (2009). ClpXP protease degrades the cytoskeletal protein, FtsZ, and modulates FtsZ polymer dynamics. Proc. Natl. Acad. Sci. U.S.A. 106, 10614–10619. 10.1073/pnas.0904886106 19541655 PMC2705540

[B26] Caplan M. R. Erickson H. P. (2003). Apparent cooperative assembly of the bacterial cell division protein FtsZ demonstrated by isothermal titration calorimetry. J. Biol.Chem. 278, 13784–13788. 10.1074/jbc.M300860200 12566454

[B27] Chen Y. Bjornson K. Redick S. D. Erickson H. P. (2005). A rapid fluorescence assay for FtsZ assembly indicates cooperative assembly with a dimer nucleus. Biophys. J. 88, 505–514. 10.1529/biophysj.104.044149 15475583 PMC1305028

[B28] Cleto S. Jensen J, V. Wendisch F. V. Lu T. K. (2016). *Corynebacterium glutamicum* metabolic engineering with CRISPR interference (CRISPRi). ACS Synth. Biol. 5, 375–385. 10.1021/acssynbio.5b00216 26829286 PMC4877668

[B29] Cohen K. A. Abeel T. Manson Mcguire A. Desjardins C. A. Munsamy V. Shea T. P. (2015). Evolution of extensively drug-resistant tuberculosis over four decades: whole genome sequencing and dating analysis of *Mycobacterium tuberculosis* isolates from. PLoS Med. 12:e1001880. 10.1371/journal.pmed.1001880 26418737 PMC4587932

[B30] Collins M. D. Hoyles L. Foster G. Sjödén B. Falsen E. (2001). *Corynebacterium capitovis* Sp. nov., from a sheep. Int. J. Syst. Evol. Microbiol. 51, 857–860. 10.1099/00207713-51-3-857 11411707

[B31] Collins M. D. Hoyles L. Lawson P. A. E. F. Robert Robson L. Foster G. (1999). Phenotypic and phylogenetic characterization of a new Corynebacterium species from dogs: description of *Corynebacterium auriscanis* Sp. nov. J. Clin. Microbiol. 37, 3443–3447. 10523531 10.1128/jcm.37.11.3443-3447.1999 PMC85662

[B32] Combs D. J. Lu Z. (2015). Sphingomyelinase D Inhibits store-operated Ca2+ entry in T lymphocytes by suppressing ORAI current. J. Gen. Physiol. 146, 161–72. 10.1085/jgp.201511359 26216860 PMC4516786

[B33] Cossart P. Jonquières R. (2000). Sortase, a Universal target for therapeutic agents against gram-positive bacteria? Proc. Natl. Acad. Sci. U.S.A. 97, 5013–5015. 10.1073/pnas.97.10.5013 10805759 PMC33977

[B34] Dajkovic A. Mukherjee A. Lutkenhaus J. (2008). Investigation of regulation of FtsZ assembly by SulA and development of a model for FtsZ polymerization. J. Bacteriol. 190, 2513–2526. 10.1128/JB.01612-07 18245292 PMC2293196

[B35] De Rose R. Tennent J. McWaters P. Chaplin P. J. Wood P. R. Kimpton W.. (2002). Efficacy of DNA vaccination by different routes of immunisation in sheep. Vet. Immunol. Immunopathol. 90, 55–63. 10.1016/S0165-2427(02)00221-0 12406655

[B36] de Souza A. P. Vale V. L. C. M. da Costa Silva I. de Oliveira Araújo B. Trindade S. C. L. de Moura-Costa F.. (2014). MAPK involvement in cytokine production in response to *Corynebacterium pseudotuberculosis* infection. BMC Microbiol. 14:230. 10.1186/s12866-014-0230-6 25179342 PMC4167526

[B37] De Zoysa A. Efstratiou A. Hawkey P. M. (2005a). Molecular characterization of diphtheria toxin repressor (dtxR) genes present in nontoxigenic *Corynebacterium diphtheriae* strains isolated in the United Kingdom. J. Clin. Microbiol. 43, 223–228. 10.1128/JCM.43.1.223-228.2005 15634975 PMC540142

[B38] De Zoysa A. Hawkey P. M. Engler K. George R. Mann G. Reilly W.. (2005b). Characterization of toxigenic Corynebacterium ulcerans strains isolated from humans and domestic cats in the United Kingdom. J. Clin. Microbiol. 43, 4377–4381. 10.1128/JCM.43.9.4377-4381.2005 16145080 PMC1234052

[B39] Demain A. L. (1990). Achievements in microbial technology. Biotechnol. Adv. 8, 291–301. 10.1016/0734-9750(90)90017-6 14545915

[B40] Demain A. L. (2000). Microbial biotechnology. Trends Biotechnol. 18, 26–31. 10.1016/S0167-7799(99)01400-6 10631778

[B41] Dias A. A. Santos L. S. Sabbadini P. S. Santos C. S. Silva Junior F. C. Napoleão F.. (2011). *Corynebacterium ulcerans* diphtheria: an emerging zoonosis in brazil and worldwide. Rev. Saúde Pública 45, 1176–1191. 10.1590/S0034-89102011000600021 22124745

[B42] Dorella F. A. Pacheco L. G. Oliveira S. C. Miyoshi A. Azevedo V. (2006). *Corynebacterium pseudotuberculosis*: microbiology, biochemical properties, pathogenesis and molecular studies of virulence. Vet. Res. 37, 201–218. 10.1051/vetres:2005056 16472520

[B43] Droppa-Almeida D. Vivas W. L. Silva K. K. Rezende A. F. Simionatto S. Meyer R.. (2016). Recombinant CP40 from *Corynebacterium pseudotuberculosis* confers protection in mice after challenge with a virulent strain. Vaccine 34, 1091–1096. 10.1016/j.vaccine.2015.12.064. 26796140

[B44] Eberhardt D. Jensen J. V. Wendisch V. F. (2014). L-Citrulline production by metabolically engineered *Corynebacterium glutamicum* from glucose and alternative carbon sources. AMB Express 4, 1–9. 10.1186/s13568-014-0085-0 26267114 PMC4883986

[B45] Efstratiou A. George R. C. (1999). Laboratory guidelines for the diagnosis of infections caused by *Corynebacterium diphtheriae* and *C. ulcerans*. World Health Organization. Commun. Dis. Public Health 2, 250–257. 10598381

[B46] Efstratiou A. Dover L. G. Holden M. T. Pallen M. Cerden A. M. Bentley S. D.. (2003). The Complete genome sequence and analysis of *Corynebacterium diphtheriae* NCTC13129. Nucleic Acids Res. 31, 6516–6523. 10.1093/nar/gkg874 14602910 PMC275568

[B47] Eggeling L. Bott M. (2005). Handbook of Corynebacterium glutamicum. Boca Raton, FL: Talyor & Francis Group.

[B48] Eikmanns B. J. Blombach B. (2014). The pyruvate dehydrogenase complex of *Corynebacterium glutamicum*: an attractive target for metabolic engineering. J. Biotechnol. 192, 339–345. 10.1016/j.jbiotec.2013.12.019 24486441

[B49] Erickson H. P. (1997). FtsZ, a tubulin homologue in prokaryote cell division. Trends Cell Biol. 7, 362–367. 10.1016/S0962-8924(97)01108-2 17708981

[B50] Erickson H. P. (2001). The FtsZ protofilament and attachment of ZipA - structural constraints on the FtsZ power stroke. Curr. Opin. Cell Biol. 13, 55–60. 10.1016/S0955-0674(00)00174-5 11163134

[B51] Fernandez-Roblas R. Adames H. Martín-de-Hijas N. Z. García Almeida D. Gadea I. Esteban J. (2009). *In vitro* activity of tigecycline and 10 other antimicrobials against clinical isolates of the genus Corynebacterium. Int. J. Antimicrob. Agents 33, 453–455. 10.1016/j.ijantimicag.2008.11.001 19153032

[B52] Fiuza M. Canova M. J. Zanella-Cléon I. Becchi M. Cozzone A. J. Mateos L. M.. (2008). From the characterization of the four serine/threonine protein kinases (PknA/B/G/L) of *Corynebacterium glutamicum* toward the role of PknA and PknB in cell division. J. Biol. Chem. 283, 18099–18112. 10.1074/jbc.M802615200 18442973

[B53] Fontaine M. C. Baird G. J. (2008). Caseous lymphadenitis. Small Rumin. Res. 76, 42–48. 10.1016/j.smallrumres.2007.12.025

[B54] Freeman V. J. Morse I. U. N. A. (1951). Studies on the virulence of bacteriophage-infected strains of *Corynebacterium diphtheriae*. J. Bacteriol. 63, 407–414. 10.1128/jb.63.3.407-414.1952 PMC169283 14927573

[B55] Frischmann A. Knoll A. Hilbert F. Zasada A. A. Kämpfer P. Busse H. J. (2012). *Corynebacterium epidermidicanis* Sp. nov., isolated from skin of a dog. Int. J. Syst. Evol. Microbiol. 62, 2194–2200. 10.1099/ijs.0.036061-0 22081710

[B56] Funke G. Bernard K. A. (2011). Coryneform gram-positive rods, in Manual of Clinical Microbiology, 10th Edn, ed Versalovic J. (Washington, DC: American Society of Microbiology), 413–442.

[B57] Funke G. Frodl R. (2008). Comprehensive study of *Corynebacterium freneyi* strains and extended and emended description of *Corynebacterium freneyi* Renaud, Aubel, Riegel, Meugnier, and Bollet 2001. J. Clin. Microbiol. 46, 638–643. 10.1128/JCM.01491-07 18077628 PMC2238093

[B58] Funke G. Frodl R. Bernard K. A. (2010). *Corynebacterium mustelae* Sp. nov., isolated from a ferret with lethal sepsis. Int. J. Syst. Evol. Microbiol. 60, 871–873. 10.1099/ijs.0.010942-0 19661496

[B59] Funke G. Lawson P. A. Collins M. D. (1998). *Corynebacterium riegelii* Sp. nov., an unusual species isolated from female patients with urinary tract infections. J. Clin. Microbiol. 36, 624–627. 9508284 10.1128/jcm.36.3.624-627.1998 PMC104597

[B60] Funke G. von Graevenitz A. Clarridge J. E. Bernard K. A. (1997). Clinical microbiology of coryneform bacteria. Clin. Microbiol. Rev. 10, 125–159. 8993861 10.1128/cmr.10.1.125 PMC172946

[B61] Galazka A. Dittmann S. (2000). The changing epidemiology of diphtheria in the vaccine era. J. Infect. Dis. 181, S2–S9. 10.1086/315533 10657184

[B62] Gamba P. Veening J. W. Saunders N. J. Hamoen L. W. Daniel R. A. (2009). Two-step assembly dynamics of the *Bacillus subtilis* divisome. J. Bacteriol. 191, 4186–4194. 10.1128/JB.01758-08 19429628 PMC2698510

[B63] Gao B. Gupta R. S. (2012). Phylogenetic framework and molecular signatures for the main clades of the phylum Actinobacteria. Microbiol. Mol. Biol. Rev. 76, 66–112. 10.1128/MMBR.05011-11 22390973 PMC3294427

[B64] Gaspar A. H. Ton-That H. (2006). Assembly of distinct pilus structures on the surface of *Corynebacterium diphtheriae*. J. Bacteriol. 188, 1526–1533. 10.1128/JB.188.4.1526-1533.2006 16452436 PMC1367254

[B65] Ginda K. Bezulska M. Ziółkiewicz M. Dziadek J. Zakrzewska-Czerwinska J. Jakimowicz D. (2013). ParA of *Mycobacterium smegmatis* co-ordinates chromosome segregation with the cell cycle and interacts with the polar growth determinant DivIVA. Mol. Microbiol. 87, 998–1012. 10.1111/mmi.12146 23289458

[B66] Goehring N. W. Beckwith J. (2005). Diverse paths to midcell: assembly of the bacterial cell division machinery. Curr. Biol. 15, 514–526. 10.1016/j.cub.2005.06.038 16005287

[B67] Gola S. Munder T. Casonato S. Manganelli R. Vicente M. (2015). The essential role of SepF in mycobacterial division. Mol. Microbiol. 97, 560–576. 10.1111/mmi.13050 25943244

[B68] Granok A. B. Benjamin P. Garrett L. S. (2002). *Corynebacterium minutissimum* bacteremia in an immunocompetent host with cellulitis. Clin. Infect. Dis. 35, e40–e42. 10.1086/341981 12145741

[B69] Guaraldi A. Hirata R. Azevedo V. (2014). *Corynebacterium diphtheriae, Corynebacterium ulcerans* and *Corynebacterium pseudotuberculosis*—general aspects, in Corynebacterium diphtheriae and Related Toxigenic Species, ed Burkovski A. (Dordrecht: Springer), 15–26.

[B70] Hadfield T. L. McEvoy P. Polotsky Y. V. Tzinserling A. A. Yakovlev A. (2000). The pathology of diphtheria. J. Infect. Dis. 181, S116–S120. 10.1086/315551 10657202

[B71] Hale C. A. De Boer P. A. (2002). ZipA Is required for recruitment of FtsK, FtsQ, FtsL, and FtsN to the septal ring in *Escherichia coli*. J. Bacteriol. 184, 2552–2556. 10.1128/JB.184.9.2552 11948172 PMC135003

[B72] Hard G. C. (1975). Comparative toxic effect of the surface lipid of *Corynebacterium ovis* on peritoneal macrophages. Infect. Immun. 12, 1439–1449. 1205621 10.1128/iai.12.6.1439-1449.1975 PMC415453

[B73] Heider S. A. Wolf N. Hofemeier A. Peters-wendisch P. Wendisch V. F. (2014). Optimization of the IPP precursor supply for the production of lycopene, decaprenoxanthin and astaxanthin by *Corynebacterium glutamicum*. Front. Bioeng. Biotechnol. 2:28. 10.3389/fbioe.2014.00028 25191655 PMC4138558

[B74] Henrique G. Lara B. Garcia M. Queico C. Leite F. Carlos A. Guazzelli A. (2011). Occurrence of *Mycobacterium* Spp. and other pathogens in lymph nodes of slaughtered swine and wild boars (*Sus scrofa*). Res. Vet. Sci. 90, 185–188. 10.1016/j.rvsc.2010.06.009 20621319

[B75] Hodgkiss W. Liston J. Goodwin T. W. Jamikorn M. (1954). The isolation and description of two marine micro-organisms with special reference to their pigment production. J. Gen. Microbiol. 11, 438–450. 10.1099/00221287-11-3-438 13221765

[B76] Hodgson K. A. Corner L. A. Rothel J. S. Radford A. J. (1992). Rational attenuation of *Corynebacterium pseudotuberculosis*: potential cheesy gland vaccine and live delivery vehicle. Infect. Immun. 60, 2900–2905. 1612756 10.1128/iai.60.7.2900-2905.1992 PMC257252

[B77] Hoelzle L. E. Scherrer T. Muntwyler J. Wittenbrink M. M. Philipp W. Hoelzle K. (2013). Differences in the antigen structures of *Corynebacterium pseudotuberculosis* and the induced humoral immune response in sheep and goats. Vet. Microbiol. 164, 359–365. 10.1016/j.vetmic.2013.02.031 23538285

[B78] Holmes R. K. (2000). Biology and molecular epidemiology of diphtheria toxin and the *Tox* gene. J. Infect. Dis. 181, S156–S167. 10.1086/315554 10657208

[B79] Holmes R. K. Barksdale L. (1970). Comparative studies with Tox + and Tox- corynebacteriophages'. J. Virol. 5, 783–794. 4193835 10.1128/jvi.5.6.783-794.1970 PMC376071

[B80] Honda E. Yanagawa R. Y. O. (1973). Deoxyribonucleic acid homologies among three immunological types of *Corynebacterium renale* (migula) ernst. Int. J. Syst. Bacteriol. 23, 226–230.

[B81] Ikeda M. Nakagawa S. (2003). The *Corynebacterium glutamicum* genome: features and impacts on biotechnological processes. Appl. Microbiol. Biotechnol. 62, 99–109. 10.1007/s00253-003-1328-1 12743753

[B82] Ishikawa S. Kawai Y. Hiramatsu K. Kuwano M. Ogasawara N. (2006). A new FtsZ-interacting protein, YlmF, complements the activity of FtsA during progression of cell division in *Bacillus subtilis*. Mol. Microbiol. 60, 1364–1380. 10.1111/j.1365-2958.2006.05184.x 16796675

[B83] James V. Carroll K. C. Funke G. Jorgensen J. H. Landry M. L. Warnock D. W. (2011). Manual of Clinical Microbiology, 10th Edn. Washington, DC: American Society for Microbiology.

[B84] Jensen J. V. Eberhardt D. Wendisch V. F. (2015). Modular pathway engineering of *Corynebacterium glutamicum* for production of the glutamate-derived compounds ornithine, proline, putrescine, citrulline, and arginine. J. Biotechnol. 214, 85–94. 10.1016/j.jbiotec.2015.09.017 26393954

[B85] Jiyoon J. L. Kim S. E. Pil Kim H. L. (2013). Adaptive evolution of *Corynebacterium glutamicum* resistant to oxidative stress and its global gene expression profiling. Biotechnol. Lett. 35, 709–717. 10.1007/s10529-012-1135-9 23288296

[B86] Jung S. I. Han M. S. Kwon J. H. Cheon C. I. Min K. H. Lee M. S. (1998). Cloning of the histidine biosynthetic genes of *Corynebacterium glutamicum*: organization and sequencing analysis of the hisA, impA, and hisF gene cluster. Biochem. Biophys. Res. Commun. 247, 741–745. 10.1006/bbrc.1998.8850 9647764

[B87] Jungwirth B. Sala C. Kohl T. A. Uplekar S. Baumbach J. Cole S. T.. (2013). High-resolution detection of DNA binding sites of the global transcriptional regulator GlxR in *Corynebacterium glutamicum*. Microbiology 159, 12–22. 10.1099/mic.0.062059-0 23103979

[B88] Kalinowski J. Bathe B. Bartels D. Bischoff N. Bott M. Burkovski A.. (2003). The complete *Corynebacterium glutamicum* ATCC 13032 genome sequence and its impact on the production of L-aspartate-derived amino acids and vitamins. J. Biotechnol. 104, 5–25. 10.1016/S0168-1656(03)00154-8 12948626

[B89] Kazmierczak M. J. Wiedmann M. Boor K. J. (2005). Alternative sigma factors and their roles in bacterial virulence. Microbiol. Mol. Biol. Rev. 69, 527–543. 10.1128/MMBR.69.4.527-543.2005 16339734 PMC1306804

[B90] Khamis A. Raoult D. La Scola B. (2004). rpoB gene sequencing for identification of Corynebacterium species. J. Clin. Microbiol. 42, 3925–3931. 10.1128/JCM.42.9.3925-3931.2004 15364970 PMC516356

[B91] Khamis A. Raoult D. La Scola B. (2005). Comparison between rpoB and 16S rRNA gene sequencing for molecular identification of 168 clinical isolates of Corynebacterium. J. Clin. Microbiol. 43, 1934–1936. 10.1128/JCM.43.4.1934 15815024 PMC1081344

[B92] Kim J. Lee M. S. (2001). Molecular cloning and analysis of the hisH gene encoding glutamine amidotransferase from *Corynebacterium glutamicum*. Korean J. Genet. 23, 121–127.

[B93] Kline K. A. Sofia Dahlberg S. F. Normark S. Henriques-Normark B. (2009). Bacterial adhesins in host-microbe interactions. Cell Host Microbe 5, 580–592. 10.1016/j.chom.2009.05.011 19527885

[B94] Koretke K. K. Lupas N. A. Warren V. P. Rosenberg M. Brown J. R. (2000). Evolution of two-component signal transduction. Mol. Biol. Evol. 17, 1956–1970. 10.1093/oxfordjournals.molbev.a026297 11110912

[B95] Krömer J. O. Bolten C. J. Heinzle E. Schröder H. Wittmann C. (2008). Physiological response of *Corynebacterium glutamicum* to oxidative stress induced by deletion of the transcriptional repressor McbR. Microbiology 154, 3917–3930. 10.1099/mic.0.2008/021204-0 19047758

[B96] Krubasik P. Sandmann G. (2000). A carotenogenic gene cluster from brevibacterium linens with novel lycopene cyclase genes involved in the synthesis of aromatic carotenoids. Mol. Gen. Genet. 263, 423–432. 10.1007/s004380051186 10821176

[B97] Krubasik P. Kobayashi M. Sandmann G. (2001a). Expression and functional analysis of a gene cluster involved in the synthesis of decaprenoxanthin reveals the mechanisms for C50 carotenoid formation. Eur. J. Biochem. 268, 3702–3708. 10.1046/j.1432-1327.2001.02275.x 11432736

[B98] Krubasik P. Takaichi S. Maoka T. Kobayashi M. Masamoto K. Sandmann G. (2001b). Detailed biosynthetic pathway to decaprenoxanthin diglucoside in *Corynebacterium glutamicum* and identification of novel intermediates. Arch. Microbiol. 176, 217–223. 10.1007/s002030100315 11511870

[B99] Kusumoto I. (2001). Glutamine metabolism : nutritional and clinical significance industrial production of L -glutamine 1. J. Nutr. 131, 2552–2555. 10.1093/jn/131.9.2552S 11533312

[B100] Kwon J. H. Chun J. Y. Lee H. S. Cheon C. I. Song E.-S. Min K. H. Lee. (2000). Cloning of the histidine biosynthetic genes from *Corynebacterium glutamicum*: organization and analysis of the hisG and hisE genes. Can. J. Microbiol. 46, 848–855. 10.1017/CBO9781107415324.004 11006846

[B101] Lan D. T. Taniguchi S. Makino S. Shirahata T. Nakane A. (1998). Role of endogenous tumor necrosis factor alpha and gamma interferon in resistance to *Corynebacterium pseudotuberculosis* infection in Mice. Microbiol. Immunol. 42, 863–870. 10037221 10.1111/j.1348-0421.1998.tb02362.x

[B102] Lee J.-Y. Na Y.-A. Kim E. Lee H.- S. Kim P. (2016). The actinobacterium *Corynebacterium glutamicum*, an industrial workhorse. J. Microbiol. Biotechnol. 26, 807–822. 10.4014/jmb.1601.01053 26838341

[B103] Lee P. C. Schmidt-Dannert C. (2003). Metabolic engineering towards biotechnological production of carotenoids in microorganisms. Appl. Microbiol. Biotechnol. 60, 1–11. 10.1007/s00253-002-1101-x 12382037

[B104] Leonard E. Ajikumar P. K. Thayer K. Xiao W. H. Tidor B.. (2010). Combining metabolic and protein engineering of a terpenoid biosynthetic pathway for overproduction and selectivity control. Proc. Natl. Acad. Sci. U.S.A. 107, 13654–13659. 10.1073/pnas.1006138107/-/DCSupplemental. 20643967 PMC2922259

[B105] Letek M. Ordóñez E. Fiuza M. Honrubia-Marcos P. Vaquera J. Gil J. A.. (2007). Characterization of the promoter region of ftsz from *Corynebacterium glutamicum* and controlled overexpression of FtsZ. Int. Microbiol. 10, 271–282. 10.2436/20.1501.01.36 18228224

[B106] Litsanov B. Brocker M. Bott M. (2012). Toward homosuccinate fermentation: metabolic engineering of *Corynebacterium glutamicum* for anaerobic production of succinate from glucose and formate. Appl. Environ. Microbiol. 78, 3325–3337. 10.1128/AEM.07790-11 22389371 PMC3346441

[B107] Lo S. Thiam I. Fall B. Ba-diallo A. Diallo O. F. Diagne R. (2015). Urinary tract infection with *Corynebacterium aurimucosum* after urethroplasty stricture of the urethra: a case report. J. Med. Case Rep. 9, 1–3. 10.1186/s13256-015-0638-0 26155836 PMC4501104

[B108] Manzella J. P. Kellogg J. A. Parsey K. S. (1995). *Corynebacterium pseudodiphtheriticum*: a respiratory tract pathogen in adults. Clin. Infect. Dis. 20, 37–40. 10.1093/clinids/20.1.37 7727667

[B109] Martaresche C. Fournier P. E. Jacomo V. Gainnier M. Boussuge A. Drancourt M. (1999). A case of *Corynebacterium pseudodiphtheriticum Nosocomial pneumonia*. Emerg. Infect. Dis. 5, 722–723. 10610204 10.3201/eid0505.990517 PMC2627726

[B110] Martin V. J. Pitera D. J. Withers S. T. Newman J. D. Keasling J. D. (2003). Engineering a mevalonate pathway in *Escherichia coli* for production of terpenoids. Nat. Biotechnol. 21, 796–802. 10.1038/nbt833 12778056

[B111] McKean S. C. Davies J. K. Moore R. J. (2007). Expression of D. phospholipase, the major virulence factor of *Corynebacterium pseudotuberculosis*, is regulated by multiple environmental factors and plays a role in macrophage death. Microbiology 153(Pt 7), 2203–2211. 10.1099/mic.0.2007/005926-0 17600064

[B112] Mentz A. Neshat A. Pfeifer-sancar Pühler, A. Rückert C. Kalinowski J. (2013). Comprehensive discovery and characterization of small RNAs in *Corynebacterium glutamicum* ATCC 13032. BMC Genomics 14:714. 10.1186/1471-2164-14-714 24138339 PMC4046766

[B113] Menzies P. I. Hwang Y.-T. Prescott J. F. (2004). Comparison of an interferon-γ to a phospholipase D enzyme-linked immunosorbent assay for diagnosis of *Corynebacterium pseudotuberculosis* infection in experimentally infected goats. Vet. Microbiol. 100, 129–137. 10.1016/j.vetmic.2004.01.012 15135521

[B114] Merhej V. Falsen E. Raoult D. (2016). *Corynebacterium timonense* Sp. nov. and *Corynebacterium massiliense* Sp. Nov., Isolated from human blood and human articular hip fluid. Int. J. Syst. Evol. Microbiol. 59, 1953–1959. 10.1099/ijs.0.005827-0. 19567562

[B115] Meyer R. Regis L. Vale V. Paule B. Carminati R. Bahia R.. (2005). *In vitro* IFN-gamma production by goat blood cells after stimulation with somatic and secreted *Corynebacterium pseudotuberculosis* antigens. Vet. Immunol. Immunopathol. 107, 249–254. 10.1016/j.vetimm.2005.05.002 15982750

[B116] Mills A. E. Mitchell R. D. Lim E. K. (2009). *Corynebacterium pseudotuberculosis* is a cause of human necrotising granulomatous lymphadenitis. Pathology 29, 231–233. 9213349 10.1080/00313029700169944

[B117] Mitrophanov A. Y. Groisman E. A. (2008). Signal integration in bacterial two-component regulatory systems. Genes Dev. 22, 2601–2611. 10.1101/gad.1700308.response 18832064 PMC2751022

[B118] Mohammadi T. van Dam V. Sijbrandi R. Vernet T. Zapun A. Bouhss A.. (2011). Identification of FtsW as a transporter of lipid-linked cell wall precursors across the membrane. EMBO J. 30, 1425–1432. 10.1038/emboj.2011.61 21386816 PMC3102273

[B119] Moreira L. O. Mattos-Guaraldi A. L. Andrade A. F. B. (2008). Novel lipoarabinomannan-like lipoglycan (CdiLAM) contributes to the adherence of *Corynebacterium diphtheriae* to epithelial cells. Arch. Microbiol. 190, 521–530. 10.1007/s00203-008-0398-y 18575847

[B120] Moura-Costa L. F. Bahia R. C. Carminati R. Vale V. L. Paule B. J. A. Portela R. W. Freire S. M.. (2008). Evaluation of the humoral and cellular immune response to different antigens of *Corynebacterium pseudotuberculosis* in canindé goats and their potential protection against *Caseous lymphadenitis*. Vet. Immunol. Immunopathol. 126, 131–141. 10.1016/j.vetimm.2008.06.013 18752855

[B121] Muckle C. A. Gyles C. L. (1983). Relation of lipid content and exotoxin production to virulence of *Corynebacterium pseudotuberculosis* in mice. Am. J. Vet. Res. 44, 1149–1153. 6870024

[B122] Naglich J. G. Metherall J. E. Russell D. W. Eidels L. (1992). Expression cloning of a diphtheria toxin receptor: identity with a heparin-binding EGF-like growth factor precursor. Cell 69, 1051–1061. 10.1016/0092-8674(92)90623-K 1606612

[B123] Neshat A. Mentz A. Rückert C. Kalinowski J. (2014). Transcriptome sequencing revealed the transcriptional organization at ribosome-mediated attenuation sites in *Corynebacterium glutamicum* and identified a novel attenuator involved in aromatic amino acid biosynthesis. J. Biotechnol. 190, 1–9. 10.1016/j.jbiotec.2014.05.033 24910972

[B124] Netzer R. Stafsnes M. H. Andreassen T. A. Goksøyr Bruheim P. Brautaset T. (2010). Biosynthetic pathway for γ-cyclic sarcinaxanthin in micrococcus luteus: heterologous expression and evidence for diverse and multiple catalytic functions of C50 carotenoid cyclases. J. Bacteriol. 192, 5688–5699. 10.1128/JB.00724-10 20802040 PMC2953688

[B125] Ochman H. Lawrence J. G. Groisman E. A. (2000). Lateral gene transfer and the nature of bacterial innovation. Nature 405, 299–304. 10.1038/35012500 10830951

[B126] Odeniyi O. A. Unuofin J. O. Adebayo-Tayo B. C. Wakil S. M. Onilude A. A. (2017). Production characteristics, activity patterns and biodecolourisation applications of thermostable laccases from *Corynebacterium efficiens* and *Enterobacter ludwigii*. J. Sci. Indus. Res. 76, 562–569.

[B127] Ogata S. Miyamoto H. Hayashida S. (1980). An investigation of the influence of bacteriophages on the bacterial flora and purification powers of activated sludge. J. Gen. Appl. Microbiol. 26, 97–108.

[B128] Olender A. (2012). Mechanisms of antibiotic resistance in *Corynebacterium* Spp. causing infections in people, in Antibiotic Resistant Bacteria - A Continuous Challenge in the New Millennium, Vol. 15, ed Pana M. (Medical University of Lublin), 387–402.

[B129] Oliveira A. Teixeira P. Azevedo M. Jamal S. B. Tiwari S. Almeida S. Silva A.. (2016). *Corynebacterium pseudotuberculosis* may be under anagenesis and biovar equi forms biovar ovis: a phylogenic inference from sequence and structural analysis. BMC Microbiol. 16:100. 10.1186/s12866-016-0717-4 27251711 PMC4890528

[B130] Ott L. Höller M. Gerlach R. G. Hensel M. Rheinlaender J. Schäffer T. E.. (2010). *Corynebacterium diphtheriae* invasion-associated protein (DIP1281) is involved in cell surface organization, adhesion and internalization in epithelial cells. BMC Microbiol. 10:2. 10.1186/1471-2180-10-2 20051108 PMC2827468

[B131] Papaventsis D. Casali N. Kontsevaya I. Drobniewski F. Cirillo D. M. Nikolayevskyy V. (2017). Whole genome sequencing of *Mycobacterium tuberculosis* for detection of drug resistance: a systematic review. Clin. Microbiol. Infect. 23, 61–68. 10.1016/j.cmi.2016.09.008 27665704

[B132] Pascual C. Lawson P. A. Farrow J. A. Gimenez M. N. Collins M. D. (1995). Phylogenetic analysis of the genus Corynebacterium based on 16S rRNA gene sequences. Int. J. Syst. Bacteriol. 45, 724–728. 7547291 10.1099/00207713-45-4-724

[B133] Peel M. M. Palmer G. G. Stacpoole A. M. Kerr T. G. (1997). Human lymphadenitis due to *Corynebacterium pseudotuberculosis*: report of ten cases from australia and review. Clin. Infect. Dis. 24, 185–191. 10.1093/clinids/24.2.185. 9114145

[B134] Pépin M. Boisramé A. Marly J. (1989). *Corynebacterium pseudotuberculosis*: biochemical properties, production of toxin and virulence of ovine and caprine strains. Ann. Vet. Res. 20, 111–115. 2930134

[B135] Pérez E. Samper S. Bordas Y. Guilhot C. Gicquel B. Martín C. (2001). An essential role for phoP in *Mycobacterium tuberculosis* virulence. Mol. Microbiol. 41, 179–187. 10.1046/j.1365-2958.2001.02500.x 11454210

[B136] Pérez-García F. Peters-Wendisch P. Wendisch V. F. (2016). Engineering *Corynebacterium glutamicum* for fast production of L-lysine and L-pipecolic Acid. Appl. Microbiol. Biotechnol. 100, 8075–8090. 10.1007/s00253-016-7682-6 27345060

[B137] Persicke M. Albersmeier A. Bednarz H. Niehaus K. Kalinowski J. Rückert C. (2015). Genome sequence of the soil bacterium *Corynebacterium callunae* type strain DSM 20147 T. Stand. Genomic Sci. 10, 1–7. 10.1186/1944-3277-10-5 26203323 PMC4510995

[B138] Pichoff S. Lutkenhaus J. (2002). Unique and overlapping roles for ZipA and FtsA in septal ring assembly in *Escherichia coli*. EMBO J. 21, 685–693. 10.1093/emboj/21.4.685 11847116 PMC125861

[B139] Pinto A. C. de Sá Caracciolo Gomes P. H. Ramos R. T. J. Barbosa S. Melo Barbosa H. P. Ribeiro A. C.. (2014). Differential transcriptional profile of *Corynebacterium pseudotuberculosis* in response to abiotic stresses. BMC Genomics 15:14. 10.1186/1471-2164-15-14 24405787 PMC3890534

[B140] Pratt S. M. Spier S. J. Carroll S. P. Vaughan B. Whitcomb M. B. W. Wilson D. (2005). Evaluation of clinical characteristics, diagnostic test results, and outcome in horses with internal infection caused by *Corynebacterium pseudotuberculosis*: 30 cases (1995-2003). J. Am. Vet. Med. Assoc. 227, 441–448. 10.2460/javma.2005.227.441 16121612

[B141] Prebble J. (1968). The carotenoids of *Corynebacterium fascians* strain 2 Y. J. Gen. Microbiol 52, 15–24.

[B142] Razak M. A. Viswanath B. (2015). Comparative studies for the biotechnological production of L -lysine by immobilized cells of wild-type *Corynebacterium glutamicum* ATCC 13032 and mutant MH 20-22 B. 3 Biotech 5, 765–774. 10.1007/s13205-015-0275-8 28324528 PMC4569635

[B143] Rebouças M. F. Portela R. W. Lima D. D. Loureiro D. Bastos B. L. Moura-Costa L. F.. (2011). Corynebacterium Pseudotuberculosis Secreted Antigen-Induced Specific Gamma-Interferon Production by Peripheral Blood Leukocytes: Potential Diagnostic Marker for Caseous Lymphadenitis in Sheep and Goats. Journal of Veterinary Diagnostic Investigation 23, 213–220. 10.1177/104063871102300204. 21398439

[B144] Riegel P. Heller R. Prevost G. Jehl F. Monteil H. (1997). *Corynebacterium durum* Sp. nov., from human clinical specimens. Int. J. Syst. Bacteriol. 47, 1107–1111. 9336915 10.1099/00207713-47-4-1107

[B145] Riegel P. Ruimy R. Briel D. D. E. Prevost G. Jehl F. Monteil H.. (1995). *Corynebacterium argentoratense* Sp. nov., from the human throat. Int. J. Syst. Bacteriol. 45, 533–537. 8590681 10.1099/00207713-45-3-533

[B146] Rocha A. A. M. C. Turk M. Z. Castro T. L. P. Abreu V. A. C. Trost E. Baumbach J. Tauch A.. (2011). Evidence for reductive genome evolution and lateral acquisition of virulence functions in two *Corynebacterium pseudotuberculosis* strains. PLoS ONE 6:e18551. 10.1371/journal.pone.0018551 21533164 PMC3078919

[B147] Rodríguez-Villalón A. Pérez-Gil J. Rodríguez-Concepción M. (2008). Carotenoid accumulation in bacteria with enhanced supply of isoprenoid precursors by upregulation of exogenous or endogenous pathways. J. Biotechnol. 135, 78–84. 10.1016/j.jbiotec.2008.02.023 18417238

[B148] Romberg L. Simon M. Erickson H. P. (2001). Polymerization of FtsZ, a bacterial homolog of tubulin. is assembly cooperative? J. Biol. Chem. 276, 11743–11753. 10.1074/jbc.M009033200 11152458

[B149] Roux V. Drancourt M. Stein A. Riegel P. Raoult D. La Scola B. (2004). Corynebacterium species isolated from bone and joint infections identified by 16S rRNA gene sequence analysis. J. Clin. Microbiol. 42, 2231–2233. 10.1128/JCM.42.5.2231-2233.2004 15131198 PMC404663

[B150] Rückert C. Albersmeier A. Al-Dilaimi A. Bednarz H. Niehaus K. Szczepanowski R. (2013). Genome sequence of the squalene-degrading bacterium *Corynebacterium terpenotabidum* TYPE STRAIN Y-11T (= DSM 44721T). Stand. Genomic Sci. 9, 505–513. 10.4056/sigs.4588337 25197436 PMC4149027

[B151] Rückert C. Albersmeier A. Al-Dilaimi A. Niehaus K. Szczepanowski R. Kalinowski J. (2012). Genome sequence of the halotolerant bacterium *Corynebacterium halotolerans* type strain YIM 70093T (= DSM 44683T). Stand. Genomic Sci. 7, 284–293. 10.4056/sigs.3236691 23408721 PMC3569386

[B152] Sandmann G. Yukawa H. (2005). Vitamin Synthesis: Carotenoids, Biotin and Pantothenate, in Handbook of Corynebacterium glutamicum, eds Eggeling L. Bott M. (Boca Raton, FL: CRC Express Journal), 399–417.

[B153] Sangal V. Hoskisson P. A. (2016). Evolution, Epidemiology and diversity of *Corynebacterium diphtheriae*: new perspectives on an old foe. Infect. Genet. Evol. 43, 364–370. 10.1016/j.meegid.2016.06.024 27291708

[B154] Sangal V. Burkovski A. Hunt A. C. Edwards B. Blom J. Hoskisson P. A. (2014). A lack of genetic basis for biovar differentiation in clinically important *Corynebacterium diphtheriae* from whole genome sequencing. Infect. Genet. Evol. 21, 54–57. 10.1016/j.meegid.2013.10.019 24200588

[B155] Saperstein S. Starr M. P. (1954). The ketonic carotenoid canthaxanthin isolated from a colour mutant of *Corynebacterium michiganense*. Biochem. J. 57, 273–275. 13172179 10.1042/bj0570273 PMC1269742

[B156] Schnell L. Mittler A.-K. Mattarei A. Tehran D. A. Montecucco C. Barth H. (2016). Semicarbazone EGA inhibits uptake of diphtheria toxin into human Cells and protects cells from intoxication. Toxins 8, 1–9. 10.3390/toxins8070221 PMC4963853 27428999

[B157] Schröder J. Maus I. Meyer K. Wördemann S. Blom J. Jaenicke S.. (2012). Complete genome sequence, lifestyle, and multi-drug resistance of the human pathogen Corynebacterium resistens DSM 45100 isolated from blood samples of a leukemia patient. BMC Genomics 13:141. 10.1186/1471-2164-13-141 22524407 PMC3350403

[B158] Schröder J. Maus I. Trost E. Tauch A. (2011). Complete genome sequence of Corynebacterium variabile DSM 44702 isolated from the surface of smear-ripened cheeses and insights into cheese ripening and flavor generation. BMC Genomics 12:545. 10.1186/1471-2164-12-545 22053731 PMC3219685

[B159] Shadnezhad A. Naegeli A. Collin M. (2016). CP40 from *Corynebacterium pseudotuberculosis* is an endo-β-N-acetylglucosaminidase. BMC Microbiol. 16:261. 10.1186/s12866-016-0884-3 27821068 PMC5100271

[B160] Sheldon M. M. Coudron P. E. (1990). Native valve endocarditis caused by an organism resembling *Corynebacterium striatum* J. Clin. Microbiol. 28, 8–10. 2298880 10.1128/jcm.28.1.8-10.1990 PMC269527

[B161] Shin N. R. Jung M. J. Kim M. S. Roh S. W. Nam Y. D. Bae J. W. (2011). *Corynebacterium nuruki* Sp. nov., isolated from an alcohol fermentation starter. Int. J. Syst. Evol. Microbiol. 61, 2430–2434. 10.1099/ijs.0.027763-0 21075904

[B162] Shyamkumar R. Muthu I. Moorthy G. Ponmurugan K. (2014). Production of L-glutamic acid with *Corynebacterium glutamicum* (NCIM 2168) and *Pseudomonas reptilivora* (NCIM 2598): a study on immobilization and reusability. Avicenna J. Med. Biotechnol. 6, 163–168. 25215180 PMC4147103

[B163] Sieger B. Bramkamp M. (2015). Interaction sites of DivIVA and RodA from *Corynebacterium glutamicum*. Front. Microbiol. 5:738. 10.3389/fmicb.2014.00738 25709601 PMC4285798

[B164] Silva J. W. Droppa-Almeida D. Borsuk S. Azevedo V. Portela R. W. Miyoshi A.. (2014). *Corynebacterium pseudotuberculosis* cp09 mutant and cp40 recombinant protein partially protect mice against *Caseous lymphadenitis*. BMC Vet. Res. 10:6. 10.1186/s12917-014-0304-6 25527190 PMC4297461

[B165] Sing A. Hogardt M. Bierschenk S. Heesemann J. (2003). Detection of differences in the nucleotide and amino acid sequences of diphtheria toxin from *Corynebacterium diphtheriae* and *Corynebacterium ulcerans* causing extrapharyngeal infections. J. Clin. Microbiol. 41, 4848–4851. 10.1128/JCM.41.10.4848-4851.2003 14532240 PMC254330

[B166] Smith K. F. Bibb L. A. Schmitt M. P. Oram D. M. (2009). Regulation and activity of a zinc uptake regulator, Zur, in *Corynebacterium diphtheriae*. J. Bacteriol. 191, 1595–1603. 10.1128/JB.01392-08 19074382 PMC2648206

[B167] Soares S. C. Silva A. Trost E. Blom J. Ramos R. Carneiro A.. (2013). The pan-genome of the animal pathogen *Corynebacterium pseudotuberculosis* reveals differences in genome plasticity between the biovar ovis and equi strains. PLoS ONE 8:e53818. 10.1371/journal.pone.0053818 23342011 PMC3544762

[B168] Soto G. E. Hultgren S. J. (1999). Bacterial adhesins: common themes and variations in architecture and assembly. J. Bacteriol. 181, 1059–1071. 9973330 10.1128/jb.181.4.1059-1071.1999 PMC93481

[B169] Starr M. P. Saperstein S. (1953). Thiamine and the carotenoid pigments of *Corynebacterium poinsettiae*. Arch. Biochem. Biophys. 43, 157–168. 13031671 10.1016/0003-9861(53)90095-2

[B170] Su H. Jiang J. Lu Q. Zhao Z. Xie T. Zhao H.. (2015). Engineering *Corynebacterium crenatum* to produce higher alcohols for biofuel using hydrolysates of duckweed (*Landoltia punctata*) as feedstock. Microb. Cell Fact. 14, 16. 10.1186/s12934-015-0199-3 25889648 PMC4324788

[B171] Sugimoto S. Yamanaka K. Nishikori S. Miyagi A. Ando T. Ogura T. (2010). AAA+ chaperone ClpX regulates dynamics of prokaryotic cytoskeletal protein FtsZ. J. Biol. Chem. 285, 6648–6657. 10.1074/jbc.M109.080739 20022957 PMC2825460

[B172] Sun Y. Guo W. Wang F. Peng F. Yang Y. (2016). Transcriptome and multivariable data analysis of *Corynebacterium glutamicum* under different dissolved oxygen conditions in bioreactors. PLoS ONE 11:e0167156. 10.1371/journal.pone.0167156. 27907077 PMC5132257

[B173] Sutherland S. S. Ellis T. M. Mercy A. R. Paton M. Middleton H. (1987). Evaluation of an enzyme-linked immunosorbent assay for the detection of *Corynebacterium pseudotuberculosis* infection in sheep. Aust. Vet. J. 64, 263–266. 3426464 10.1111/j.1751-0813.1987.tb15953.x

[B174] Swierczynski A. Ton-That H. (2006). Type III pilus of corynebacteria: pilus length is determined by the level of its major pilin subunit. J. Bacteriol. 188, 6318–6325. 10.1128/JB.00606-06 16923899 PMC1595371

[B175] Tao L. Yao H. Cheng Q. (2007). Genes from a Dietzia Sp. for synthesis of C40 and C50 β-cyclic carotenoids. Gene 386, 90–97. 10.1016/j.gene.2006.08.006 17008032

[B176] Tauch A. Sandbote J. (2014). The Prokaryotes: Actinobacteria. eds Rosenberg E. DeLong E. F. Lory S. Stackebrandt E. Thompson F. (Berlin; Heidelberg: Springer Berlin Heidelberg), 239–277.

[B177] Tiwari S. da Costa M. P. Almeida S. Hassan S. S. Jamal S. B. Oliveira A.. (2014). *C. pseudotuberculosis* phop confers virulence and may be targeted by natural compounds. Integr. Biol. 6, 1088–1099. 10.1039/c4ib00140k 25212181

[B178] Tong J. Han Q. Wang S. Su Z. Zheng D. Shen P.. (2012). *Corynebacterium pyruviciproducens*, as an immune modulator, can promote the activity of macrophages and up-regulate antibody response to particulate antigen. Exp. Biol. Med. 237, 1322–1330. 10.1258/ebm.2012.012181 23239443

[B179] Tong J. Liu C. Summanen P. H. Xu H. Finegold S. M. (2010). *Corynebacterium pyruviciproducens* Sp. nov., a pyruvic acid producer. Int. J. Syst. Evol. Microbiol. 60, 1135–1140. 10.1099/ijs.0.011783-0 19666798

[B180] Ton-That H. Schneewind O. (2003). Assembly of pili on the surface of *Corynebacterium diphtheriae*. Mol. Microbiol. 50, 1429–1438. 10.1046/j.1365-2958.2003.03782.x 14622427

[B181] Ton-That H. Marraffini L. A. Schneewind O. (2004). Protein sorting to the cell wall envelope of gram-positive bacteria. Biochim. Biophys. Acta 1694, 269–278. 10.1016/j.bbamcr.2004.04.014 15546671

[B182] Trost E. Al-Dilaimi A. Papavasiliou P. Schneider J. Viehoever P. Burkovski A. Soares C. S.. (2011). Comparative analysis of two complete *Corynebacterium ulcerans* genomes and detection of candidate virulence factors. BMC Genomics 12:383. 10.1186/1471-2164-12-383 21801446 PMC3164646

[B183] Trost E. Blom J. De Castro Soares S. Huang I.-H. Al-Dilaimi A. Schröder J.. (2012). Pangenomic study of *Corynebacterium diphtheriae* that provides insights into the genomic diversity of pathogenic isolates from cases of classical Diphtheria, Endocarditis, and Pneumonia. J. Bacteriol. 194, 3199–3215. 10.1128/JB.00183-12 22505676 PMC3370855

[B184] Trost E. Ott L. Schneider J. Schröder J. Jaenicke S. Goesmann A.. (2010). The complete genome sequence of *Corynebacterium pseudotuberculosis* FRC41 isolated from a 12-year-old girl with necrotizing lymphadenitis reveals insights into gene-regulatory networks contributing to virulence. BMC Genomics 11:728. 10.1186/1471-2164-11-728 21192786 PMC3022926

[B185] Vale V. L. da Costa Silva M. de Souza A. P. Trindade S. C. de Moura-Costa E. K. Dos Santos-Lima N. I.. (2016). Humoral and cellular immune responses in mice against secreted and somatic antigens from a *Corynebacterium pseudotuberculosis* attenuated strain: immune response against a *C. pseudotuberculosis* strain. BMC Vet. Res. 12:195. 10.1186/s12917-016-0811-8 27608632 PMC5017044

[B186] Varela C. A. Baez M. E. Agosin E. (2004). Osmotic stress response: quantification of cell maintenance and metabolic fluxes in a lysine-overproducing strain of *Corynebacterium glutamicum*. Appl. Environ. Microbiol. 70, 4222–4229. 10.1128/AEM.70.7.4222-4229.2004 15240305 PMC444767

[B187] Vívian D. Soares A. A. S. C. Santos P. A. C. A. R. Magalhães A. A. C. Faria C. J. Barbosa E. (2012). Reannotation of the *Corynebacterium diphtheriae* NCTC13129 genome as a new approach to studying gene targets connected to virulence and pathogenicity in diphtheria. Open Access Bioinformatics 4, 1–13. 10.2147/OAB.S25500

[B188] Volante A. Alonso J. C. (2015). Molecular anatomy of ParA-ParA and ParA-ParB interactions during plasmid partitioning. J. Biol. Chem. 290, 18782–18795. 10.1074/jbc.M115.649632 26055701 PMC4513133

[B189] Wagner K. S. White J. M. Crowcroft N. S. De Martin S. Mann G. Efstratiou A. (2010). Diphtheria in the United Kingdom, 1986-2008: the increasing role of *Corynebacterium ulcerans*. Epidemiol. Infect. 138, 1519–1530. 10.1017/S0950268810001895 20696088

[B190] Wagner K. S. White J. M. Lucenko I. Mercer D. Crowcroft N. S. Neal S.. (2012). Diphtheria in the postepidemic period, Europe, 2000-2009. Emerg. Infect. Dis. 18, 217–225. 10.3201/eid1802.110987 22304732 PMC3310452

[B191] Wattam A. R. J. Davis J. Assaf R. Brettin T. Bun C. Conrad N.. (2017). Improvements to PATRIC, the all-bacterial bioinformatics database and analysis resource. Nucleic Acids Res. 45, 535–542. 10.1093/nar/gkw1017 27899627 PMC5210524

[B192] Wattam A. R. Abraham D. Dalay O. Disz T. L. Driscoll T. Gabbard J. L. Gillespie J. J.. (2014). PATRIC, the bacterial bioinformatics database and analysis resource. Nucleic Acids Res. 42, D581–D591. 10.1093/nar/gkt1099 24225323 PMC3965095

[B193] Wattiau P. Janssens M. Wauters G. (2000). *Corynebacterium simulans* Sp. nov., a nonlipophilic, fermentative corynebacterium. Int. J. Syst. Evol. Microbiol. 50, 347–353. 10.1099/00207713-50-1-347 10826822

[B194] Wendisch V. Jorge J. M. P Pérez-García, F. Sgobba E. (2016). Updates on industrial production of amino acids using *Corynebacterium glutamicum*. World J. Microbiol. Biotechnol. 32:10. 10.1007/s11274-016-2060-1 27116971

[B195] Williamson L. H. (2001). *Caseous lymphadenitis* in small ruminants. Vet. Clin. N. Am. Food Anim. Pract. 17, 359–371. 10.1016/S0749-0720(15)30033-5 11515406

[B196] Windsor P. A. Bush R. D. (2016). *Caseous lymphadenitis*: present and near forgotten from persistent vaccination? Small Rumin. Res. 142, 6–10. 10.1016/j.smallrumres.2016.03.023

[B197] Wong J. S. J. Lois S. M. Ho C. P. Anderson T. P. E. Lau O. C.. (2010). *Corynebacterium accolens*-associated *Pelvic osteomyelitis*. J. Clin. Microbiol. 48, 654–655. 10.1128/JCM.00818-09 20032254 PMC2815633

[B198] Wu L. J. Errington J. (2011). Nucleoid occlusion and bacterial cell division. Nat. Rev. Microbiol. 10, 8–12. 10.1038/nrmicro2671 22020262

[B199] Xu M. Rao Z. Dou W. Yang J. Jin J. Xu Z. (2012). Site-directed mutagenesis and feedback-resistant N-acetyl-L-glutamate kinase (NAGK) increase *Corynebacterium crenatum* L-arginine production. Amino Acids 43, 255–266. 10.1007/s00726-011-1069-x 21901472

[B200] Yanagawa R. Otsuki K. Tokui T. (1968). Electron microscopy of fine structure of *Corynebacterium renale* with special reference to pili. Jpn. J. Vet. Res. 16, 2–18. 5304169

